# Synthesis of Iron(II) Clathrochelate-Based Poly(vinylene sulfide) with Tetraphenylbenzene Bridging Units and Their Selective Oxidation into Their Corresponding Poly(vinylene sulfone) Copolymers: Promising Materials for Iodine Capture

**DOI:** 10.3390/polym14183727

**Published:** 2022-09-07

**Authors:** Noorullah Baig, Suchetha Shetty, Sameh S. Habib, Ali A. Husain, Saleh Al-Mousawi, Bassam Alameddine

**Affiliations:** 1 Department of Mathematics and Natural Sciences, Gulf University for Science and Technology, Hawally 32093, Kuwait; 2 Functional Materials Group, GUST, Hawally 32093, Kuwait; 3 Department of Chemistry Kuwait City, Kuwait University, P.O. Box 12613, Safat 13060, Kuwait

**Keywords:** metalorganic polymer, poly(vinylene sulfide), poly(vinylene sulfone), one-pot synthesis, click-reaction, iodine adsorption

## Abstract

The development of a simple and efficient synthetic methodology to engineer functional polymer materials for gas adsorption is necessary due to its relevance for various applications. Herein, we report the synthesis of metalorganic poly(vinylene sulfide) copolymers CTP1-3 with iron(II) clathrochelate of various side groups connected by tetraphenylbenzene units. CTP1-3 were subsequently oxidized into their respective poly(vinylene sulfone) copolymers CTP4-6 under green reaction conditions. The target copolymers CTP1-6 were characterized using various instrumental analysis techniques. Examination of the iodine adsorption properties of the copolymers revealed high iodine uptake properties, reaching 2360 mg g^−1^ for CTP2, and whose reusability tests proved its efficient regeneration, thus proving the importance of iron(II) clathrochelate polymers in iodine capture.

## 1. Introduction

The global demand for energy is drastically increasing due to the surge in world population and economic development. Energy consumed in 2021 has achieved a historical world record [1] and it is estimated that by 2040 the worldwide demand for primary energy will exceed 800 quadrillion British thermal units [2]. Since the industrial revolution, fossil fuels have always been employed as the chief energy source, accounting for ~82% of the total energy consumed in 2021 [1]. This major reliance on fossil fuel combustion to produce energy entails serious impacts on the environment caused by the emission of various harmful gases into the atmosphere, namely, carbon dioxide, which contributes to global warming, and nitrogen and sulfur oxides (i.e., NOx and SOx), which produce acid rain and release particulate matter (PM) that leads to smog formation, thus, bringing about serious environmental and health complications [3,4,5,6]. These risks have consequently led to global initiatives and extensively funded research programs to find alternative energy sources with properties that overcome the hurdles encountered with fossil fuels: namely, renewability, efficiency, and zero- or low- greenhouse gases emission. Interestingly, renewable energy resources continue to grow strongly every year, showing a ~17% increase in 2021, and thus accounting for ~13% of the total power generation during that year [1].

Nuclear power is amongst the most important sources to produce large-scale renewable energy in the world [7]. Ideally, nuclear energy is rather safe, economically competitive, and relatively clean as it does not release greenhouse gases into the atmosphere [8,9]. Nevertheless, increasing the production of nuclear energy requires overcoming some major concerns, namely, those related to improving the safety operations and protocols in nuclear power plants, on the one hand, and disposing of nuclear waste more efficiently, on the other hand [10,11]. Nuclear accidents such as those that occurred in Chernobyl in 1986 and Fukushima in 2011 remain remembered as extremely perilous because of the devastating effects of the blasts in the respective areas, along with the emission of myriad radioactive gases: namely, strontium (^90^Sr), technetium (^99^Tc), cesium (^137^Cs), and various volatile radionuclides such as ^3^H,^14^C, ^85^Kr, ^123^I, ^125^I, and ^127−140^I [12,13,14]. Amongst the latter iodine isotopes, both ^129^I and ^131^I with half-lives of 8 days and 15.7 million years, respectively, are considered to be the most hazardous waste byproducts of the nuclear fission process [15], causing severe chronic health effects on the metabolic and reproductive systems [16,17]. As a matter of fact, the Fukushima nuclear power plant incident has released huge quantities of radiological pollutants, among others, ^131^I, ^134^Cs, and ^137^Cs, which engender several complications, such as thyroid cancers [18,19], low birth weight or preterm birth [20,21], and malformed fetuses [22]. Consequently, in view of its threat to both human health and the environment, several materials have been developed to capture radioactive iodine species effectively both from solutions and gases [23,24,25] by using zeolites [13,26,27], carbon materials [28,29,30,31], mesoporous silica [32,33], MOFs [34,35], and molecular sieves [36].

Iron (II) clathrochelates are promising materials due to their intricate structure, robust nature, easy synthesis, post-functionalization, and versatile applications [37,38,39,40,41,42]. Several materials derived from iron(II) clathrochelate moieties have been synthesized and their adsorption properties were tested against various gases and dyes [37,43,44,45,46,47]. This work discloses the synthesis of three metalorganic poly(vinylene sulfide) copolymers CTP1-3 in high yield under mild condition using a diboronic acid synthon, made from a catalyst-free thiol-yne click-reaction, and reacted with iron(II) chloride and various 1,2-dioximes. The resulting metalorganic copolymers CTP1-3 were subsequently oxidized into their corresponding poly(vinylene sulfone) derivatives CTP4-6 using environmentally friendly reaction conditions before testing the iodine capture properties of copolymers CP1-6.

## 2. Materials

All the reactions were carried out under an inert atmosphere of dry argon. All the chemical reagents were used without further purification as purchased unless otherwise specified. The required 4-Mercaptophenylboronic acid, 1,2-Cyclohexanedione dioxime, Anti-Diphenylglyoxime, FeCl_2_, H_2_O_2,_ AcOH, and I_2_ were purchased from Merck (Darmstadt, Germany). Compound 1 was synthesized following a reported procedure [48,49]. Anhydrous solvents, namely tetrahydrofuran (THF), hexane, ethanol, dichloromethane (DCM), methanol, and chloroform (CHCl_3_), were procured from Loba Chemie (Mumbai, India), Fisher Scientific (New Hampshire, United States), and Merck (Darmstadt, Germany). All the solvents were dried over molecular sieves and deoxygenated by bubbling with dry argon gas for 30 min.

### 2.1. Characterization Techniques

Thin layer chromatography (TLC) was performed on aluminum sheets coated with silica gel 60 F254 and revealed using a UV lamp. NMR (^1^H: 600 MHz, ^13^C: 150 MHz) spectra were recorded using a Bruker BioSpin GmbH 600 MHz spectrometer using DMSO-d_6_, CDCl_3_, and CD_2_Cl_2_ as a solvent with the chemical shifts (*δ*) given in ppm and referenced to tetramethylsilane (TMS). Electron impact high-resolution mass spectra (EI-HRMS) were recorded on a Thermo Scientific DFS system with a standard PFK (perfluorokerosene) as a lock mass. The analyzed data were converted to accurate mass employing the Xcalibur accurate mass calculation software. UV-Vis spectra were recorded using a Shimadzu UV1800 spectrophotometer. FTIR spectra were recorded on an FT/IR-6300 type A instrument using a KBr matrix. Thermogravimetric analysis (TGA) was recorded on a Shimadzu TGA-60H (Kyoto, Japan) analyzer and used to measure the thermal stability of composites. TGA was carried out from room temperature to 800 °C. The heating rate was kept at 10 °C/min under inert atmosphere using pure nitrogen. X-ray photoelectron spectroscopy (XPS) data were recorded with a Thermo ESCALAB 250 Xi using a monochromatic Al K-radiation source (1486.6 eV) with a spot size of 850 µm. Spectra acquisition and processing were carried out using the software Thermo Advantage Version 4.87. The base pressure in the XPS analysis chamber was in the range 10^−10^ to 10^−9^ torr. The analyzer was operated with pass energy of 20 eV, dwell time of 50 min, and with a step size of 0.1 eV. Agilent Gel Permeation Chromatography (GPC/SEC) equipped with two columns (PL mixed-C) and calibrated against twelve monodisperse polystyrene (PS) standards, using THF as eluent at a flow rate of 1.0 mL min^−1^, was employed to determine the relative weight-average (Mw) and number-average (Mn) molecular weights, and polydispersity index (Đ = M_w_/M_n_) of all the reported polymers.

### 2.2. Synthesis

#### 2.2.1. Synthesis of ((((2′,3′-diphenyl-[1,1′:4′,1″-terphenyl]-4,4″-diyl)bis(ethene-2,1-diyl))bis(sulfanediyl))bis(4,1-phenylene))diboronic Acid (TBM)

3′,6′-bis(4-ethynylphenyl)-1,1′:2′,1″-terphenyl 3 (670 mg, 1.55 mmol, 1 eq.) and 4-mercaptophenylboronic acid 4 (477 mg, 3.1 mmol, 2 eq) in THF (30 mL) were charged in a Schlenk tube under argon. The reaction mixture was heated at 30 °C for 15 h and the resulting solution was added dropwise to 250 mL of hexane while stirring. The precipitate was filtered and washed with hexane (20 mL). Light yellow solid (1.05 g, 91%). ^1^H-NMR (600 MHz, DMSO-d_6_, ppm): *δ* 7.82–7.77 (*m*, 4H, ArH), 7.53–7.48 (*m*, 2H, ArH), 7.43 (*d*, 2H, *J* = 8.1 Hz, ArH), 7.36–7.34 (*m*, 6H, ArH), 7.17–7.15 (*m*, 2H, ArH), 7.09–7.07 (*m*, 2H, ArH), 6.97 (*br*, 8H, ArH), 6.89–6.86 (*m*, 6H, alkene-CH, and ArH); ^13^C-NMR (150 MHz, DMSO-d_6_, ppm): *δ* 140.11, 139.43, 137.64, 137.01, 135.03, 134.96, 134.94, 134.03, 133.41, 131.79, 131.04, 129.55, 127.79, 127.38, 126.88, 125.60, 124.36, 121.66, 116.42; FTIR (KBr, cm^−1^): 3204 (O-H str), 3023 (Ar-CH str), 1606 (C=C str), 1346 (B-O str), 1184 (B-C str), 825 (C-H ben), 699 (Alkene C=C str); EI-HRMS: *m*/*z* calculated for M^•+^ C_46_H_36_B_2_O_4_S_2_ 738.2241 found 738.2241.

#### 2.2.2. Synthesis of Copolymer CTP1 (Procedure A)

A solution of TBM (206 mg, 0.28 mmol, 1 eq.), butyl dioxime BD (168 mg, 0.84 mmol, 3 eq.), and iron (II) chloride (FeCl_2_, 35 mg 0.28 mmol, 1 eq.) in 11 mL of degassed chloroform was refluxed under argon for five days and the solvent was evaporated under reduced pressure. The resulting mixture was dissolved in DCM and extracted with a saturated solution of NaHCO_3_ (20 mL × 2). The combined organic layer was washed with deionized water (50 mL × 3), concentrated, and the desired product isolated by precipitation in DCM/methanol. Brick-red solid (333 mg, 91%). GPC (THF); M_w_ (g mol^−1^): 9554 Mn (g mol^−1^): 5381, Đ: 1.77; ^1^H-NMR (600 MHz, CD_2_Cl_2_, ppm): *δ* 7.72 (*br*, 4H, ArH), 7.58–7.41 (*brm*, 8H, ArH), 7.24–7.12 (*brm*, 6H, ArH), 7.01–6.86 (*brm*, 14H, alkene-CH, and ArH), 2.86 (*brs*, 12H, CH_2_), 1.58 (*brs*, 12H, CH_2_), 1.38 (*brs*, 12H, CH_2_), 0.94 (*brm*, 18H, CH_3_); ^13^C-NMR (150 MHz, CD_2_Cl_2_, ppm): *δ* 157.77, 141.05, 140.71, 135.27, 134.22, 133.16, 132.39, 132.13, 130.76, 130.40, 129.83, 129.63, 129.50, 128.52, 127.48, 127.10, 126.26, 125.68, 29.75, 27.64, 23.12, 14.23; FTIR (KBr, cm^−1^): 2957 (Aliphatic C-H str), 1604 (C=C str), 1460 (Aliphatic C-H ben) 1399 (B-O str), 1182 (B-C str), 824 (Ar-C-H ben), 694 (Alkene C=C str).

#### 2.2.3. Synthesis of Copolymer CTP2

CTP2 was prepared following procedure A with: TBM (206 mg, 0.28 mmol, 1 eq.), 1,2-cyclohexanedione dioxime CD (119 mg, 0.84 mmol, 3 eq.), FeCl_2_ (35 mg 0.28 mmol, 1 eq.) and degassed chloroform (11 mL). Red solid (293 mg, yield = 93%); FTIR (KBr, cm^−1^): 2939 (Aliphatic C-H str), 1601 (C=C str), 1442 (Aliphatic C-H ben) 1383 (B-O str), 1194 (B-C str), 817 (Ar-C-H ben), 691 (Alkene C=C str).

#### 2.2.4. Synthesis of Copolymer CTP3

CTP3 was prepared following procedure A with: TBM (206 mg, 0.28 mmol, 1 eq.), anti-diphenylglyoxime PD (201 mg, 0.84 mmol, 3 eq.), FeCl_2_ (35 mg 0.28 mmol, 1 eq.) and degassed chloroform (11 mL). Red solid (360 mg, yield = 90%); FTIR (KBr, cm^−1^): 3058 (Ar-C-H str), 1606 (C=C str), 1446 (Aliphatic C-H ben) 1359 (B-O str), 1195 (B-C str), 817 (Ar-C-H ben), 690 (Alkene C=C str).

#### 2.2.5. Synthesis of CTP4 (Procedure B)

To a stirring solution of CTP1 (86 mg, 0.065 mmol) in acetic acid (4 mL) was added dropwise 1.5 mL of a 30 wt% aqueous solution H_2_O_2_. The reaction mixture was stirred at 90 °C for 10 min. The precipitate was filtered off and washed with deionized water (20 mL) and methanol (2 mL) then dried under vacuum to yield CTP4 as a red solid (84 mg, 93%). GPC (THF); M_w_ (g mol^−1^): 8436 Mn (g mol^−1^): 5126, Đ: 1.64; ^1^H-NMR (600 MHz, CD_2_Cl_2_, ppm): *δ* 7.91–6.88 (bm, 32H, alkene-CH, and ArH), 2.86 (*brs*, 12H, CH_2_), 1.61 (*brs*, 12H, CH_2_), 1.39 (*brs*, 12H, CH_2_), 0.94 (*brm*, 18H, CH_3_); ^13^C-NMR (150 MHz, CD_2_Cl_2_, ppm): *δ* 158.02, 141.05, 140.43, 135.26, 133.20, 132.07, 130.70, 130.65, 129.81, 129.62, 128.54, 127.60, 126.44, 125.67, 29.75, 27.65, 23.03, 14.23; FTIR (KBr, cm^−1^): 2947 (Aliphatic C-H str), 1608 (C=C str), 1460 (Aliphatic C-H ben) 1395 (B-O str), 1312 (S=O str), 1182 (B-C str), 1130 (S=O str), 812 (Ar-C-H ben), 693 (Alkene C=C str).

#### 2.2.6. Synthesis of CTP5

CTP5 was prepared following procedure B with: CTP2 (60 mg, 0.042 mmol), acetic acid (3 mL), 1.25 mL of 30 wt% aqueous solution H_2_O_2_. The reaction mixture was stirred at 90 °C for 30 min. Red solid (61 mg, 98%); FTIR (KBr, cm^−1^): 2944 (Aliphatic C-H str), 1608 (C=C str), 1488 (Aliphatic C-H ben) 1393 (B-O str), 1309 (S=O str), 1198 (B-C str), 1146 (S=O str), 810 (Ar-C-H ben), 699 (Alkene C=C str).

#### 2.2.7. Synthesis of CTP6

CTP6 was prepared following procedure B with: CTP3 (80 mg, 0.056 mmol), acetic acid (4 mL), 1.5 mL of 30 wt% aqueous solution H_2_O_2_. The reaction mixture was stirred at 90 °C for 30 min. Red solid (81 mg, 97%); FTIR (KBr, cm^−1^): 3053 (Ar-C-H str), 1603 (C=C str), 1393 (B-O str), 1316 (S=O str), 1219 (B-C str), 1145 (S=O str), 839 (Ar-C-H ben), 693 (Alkene C=C str).

### 2.3. Iodine Adsorption Studies of CTP1-6

A 25 mg sample of a given copolymer (CTP1-6) and iodine flakes were placed in two connected and closed pre-weighed bottles. The vessel was degassed and maintained at 80 °C. The iodine uptake was measured at different time intervals and the amount of iodine adsorbed was calculated using the following equation [50]:

M_2_ − M_1_/M_1_ × 100% (100 wt% = 1000 mg g^−1^), (1)

where M_2_ and M_1_ are the masses of the given copolymer after and before iodine uptake, respectively.

#### Adsorption Equilibrium and Kinetics

The adsorption kinetics were explored using the pseudo-first-order and pseudo-second-order models, expressed respectively in Equations (2) and (3) below [50]:

ln(*Q*_e_ − *Q_t_*) = ln *Q*_e_ − *K*_1_*t* (2)

t/*Q*_t_ = t/*Q*_e_ + 1/K_2_*Q*_e_^2^ (3)

where *Q_t_* (mg g^−1^) and *Q*_e_ (mg g^−1^) denote the amounts of iodine adsorbed per gram adsorbent at time *t* and at equilibrium, respectively. *K* _1_ and *K* _2_ (mg g^−1^ h^−1^) represent the rate constants of the pseudo-first-order model and pseudo-second-order model, respectively.

## 3. Results

### 3.1. Synthesis of TBM

The diethynyl tetraphenylbenzene derivative 3 was synthesized following a reported procedure [48,51,52] and its structure was confirmed by ^1^H- and ^13^C- nuclear magnetic resonance (NMR), high-resolution mass spectrometry (EI-HRMS), FTIR spectroscopy and single-crystal XRD (synthetic procedures (i) and (ii), in addition to Figures S1, S5, S10 and S12 in Supplementary Materials).

**Scheme 1:**
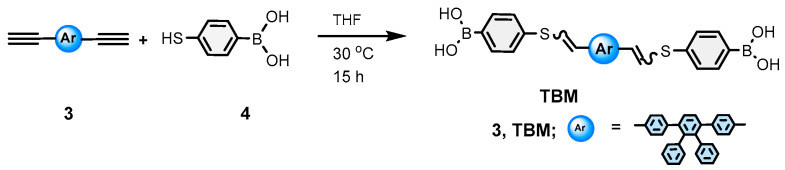
Synthesis of comonomer TBM.

Scheme 1 reveals the reaction conditions to make the diboronic acid synthon TBM employing a versatile metal-free thiol-yne click reaction of 3′,6′-bis(4-ethynylphenyl)-1,1′:2′,1′’-terphenyl 3 with 4-mercaptophenylboronic acid 4 in THF at 30 °C for 15 h [53,54]. The structure of the desired synthon TBM was confirmed by ^1^H- and ^13^C- nuclear magnetic resonance (NMR), high-resolution mass spectrometry (EI-HRMS), and FTIR spectroscopy (Figures S2, S6, S11 and S12 in Supplementary Materials).

### 3.2. Synthesis of Copolymers CTP1-3

**Scheme 2:**
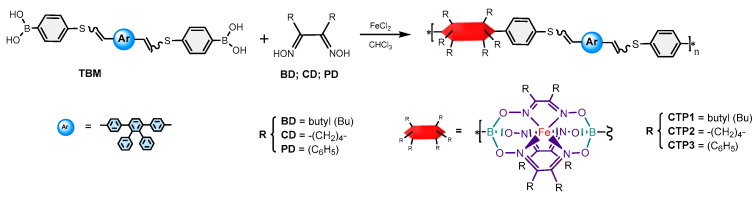
Synthesis of copolymers CTP1-3.

As noted from Scheme 2, copolymers CTP1-3 were successfully made from the one-pot complexation reaction of iron(II) chloride with the specially designed diboronic acid comonomer synthon TBM with each of butyl dioxime BD, 1,2-cyclohexanedione dioxime CD, and anti-diphenylglyoxime PD.

**Figure 1:**
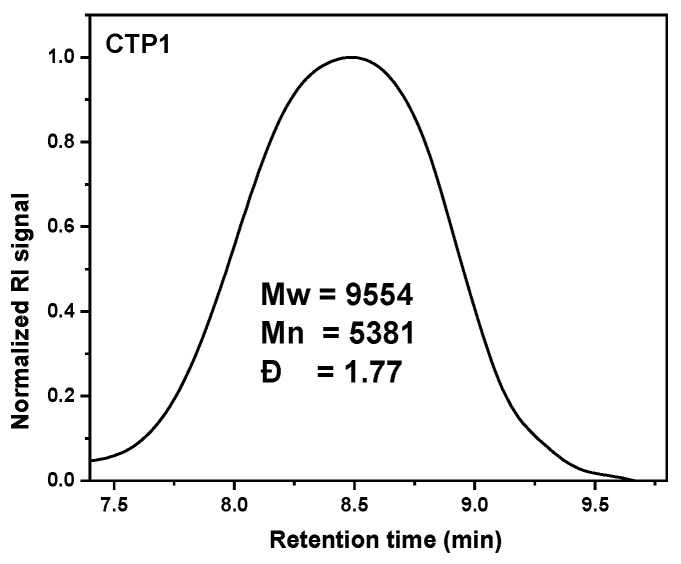
Normalized GPC chromatogram of CTP1.

Table 1 summarizes the attempts carried out to optimize the copolymerization reaction conditions. Synthesis of CTP1 was first tried by reacting a 0.05 M concentration of TBM and three equivalents of BD in presence of one equivalent of FeCl_2_ in refluxing chloroform for one day, which afforded the desired copolymer in 67% yield and whose GPC analysis revealed the formation of short chains weight-average molecular weight M_w_ ≈ 10 KDa (Table 1, entry 1). Additionally, doubling the reaction time led to a lower yield (~30%) and resulted in the formation of polymers with M_w_ ≈ 40 KDa but which suffer from a large polydispersity Ð = 4.6 (Table 1, entry 2). Therefore, to improve the solubility of all the species during the reaction, the concentration of the comonomers was further diluted by doubling the amount of solvent in the medium, thus, reaching a molar concentration of 0.025 M for TBM, and the reaction time was also increased to three days instead of one, which afforded a slight improvement in the reaction yield to 45% of the isolated copolymer CTP1 revealing a molar mass M_w_ ≈ 10 KDa with a polydispersity Ð = 2.8 (Table 1, entry 3). Further dilution of the comonomers by a factor of 2.5 (i.e., 0.01 M of TBM) resulted only in a slight improvement in both the reaction yield (52%) and molar mass (Table 1, entry 4). It is worthwhile to mention that oligomers and starting materials were detected in all the previous copolymerization reactions. Therefore, the reaction time was increased to five days to ensure complete reaction where pure CTP1 was obtained in 91% yield and a weight-average molecular weight M_w_ ≈ 9.5 KDa with Ð ≈ 1.8 from the reaction of a 0.025 M concentration of TBM, three equivalents of BD, and one equivalent of FeCl_2_ in refluxing chloroform (Figure 1 and Table 1, entry 5). Similar reaction conditions were utilized in the copolymerization of TBM in the presence of an equimolar amount of FeCl_2_ with three equivalents of either CD or PD, which afforded CTP2 and CTP3 in 93% and 90% yields, respectively, as highly insoluble copolymers.

The structure and high purity of CTP1 were confirmed by GPC analyses and ^1^H- and ^13^C-NMR spectroscopy (Figure 1 and Figure 2, and Figures S3 and S7 in Supplementary Materials). In addition, target copolymers CTP1-3 were characterized by FTIR, XPS, and TGA (Figure 3, Figure 4 and Figure 7 and Figures S13, S14, S18 and S19 in Supplementary Materials).

**Figure 2:**
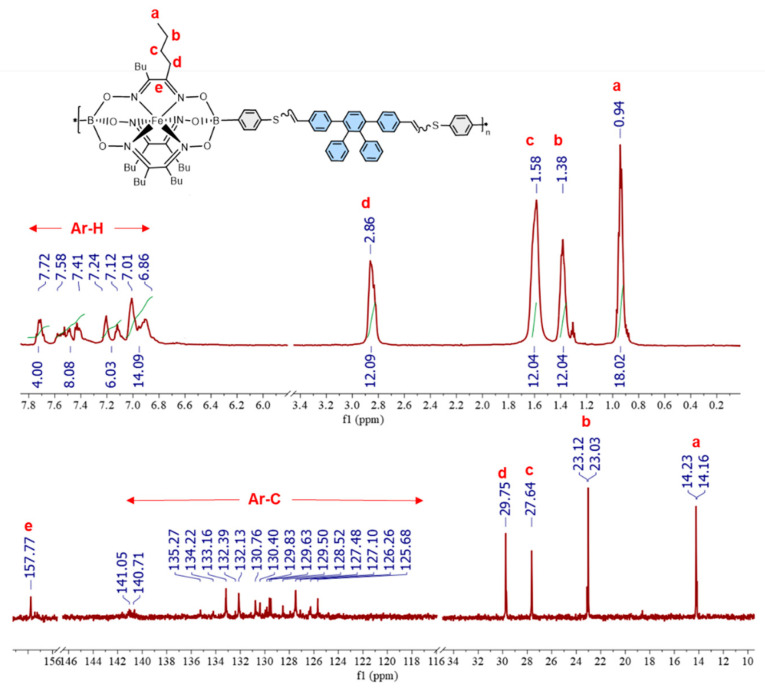
Representative ^1^H- (**upper**) and ^13^C-NMR (**lower**) spectra of CTP1.

Figure 2 depicts the ^1^H- and ^13^C-NMR spectra of copolymer CTP1 confirming its structure as proven by the presence of all the desired peaks. The aromatic protons and carbons of CTP1 were detected in the ranges 6.8–7.7 and 125.6–141.0 ppm, respectively. It is noteworthy that all the characteristic proton peaks of the butyl group were depicted at 0.9, 1.3, 1.5, and 2.8 ppm, whereas those carbon peaks of the latter group were identified at 14.2, 23.1, 27.6, and 29.7 ppm (peaks labeled a–d in Figure 2). Furthermore, the distinctive C=N carbon in the clathrochelate moiety of CTP1 was detected at 157.7 ppm, which supports the formation of the desired product (peak labeled e in Figure 2).

**Figure 3:**
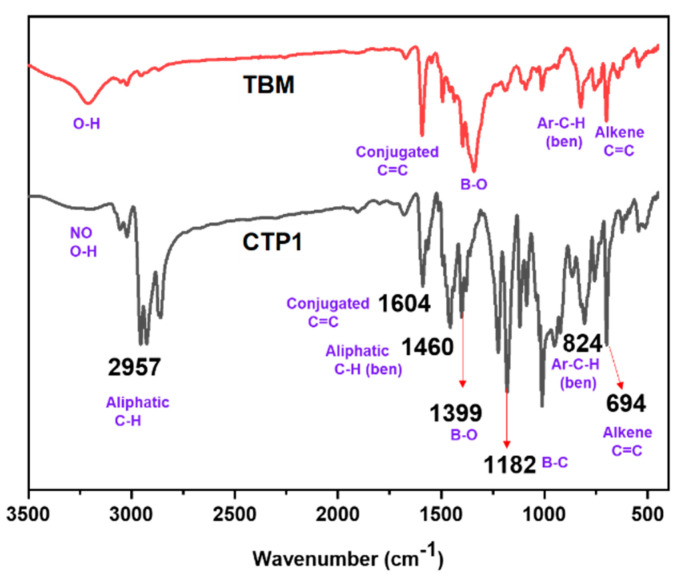
Comparative FTIR spectrum of TBM (**upper**) and CTP1 (**lower**).

Figure 3 portrays the comparative FTIR absorption spectra for both comonomer TBM and target copolymer CTP1. The characteristic stretching vibrations of the hydroxyl groups (O-H) were detected at ~3212 cm^−1^ for TBM, which disappear in the spectrum of copolymer CTP1. It is noteworthy that the absorption bands identified at ~2957 cm^−1^ and ~1460 cm^−1^ correspond to the distinctive aliphatic C-H stretching and bending vibrations, respectively, and which clearly indicate the presence of the butyl group in CTP1. In addition, the fingerprint stretching vibrations [55] were confirmed for each of the conjugated C=C (~1604 cm^−1^), B-O (1399 cm^−1^), B-C aromatic (1182 cm^−1^), and C-H (824 cm^−1^) and alkene C=C (694 cm^−1^) bending vibration peaks, which further supports the formation of target copolymer CTP1. Similarly, FTIR absorption spectra of target copolymers CTP2-3 reveal their distinctive stretching and bending vibration peaks that corroborate their successful formation (Figures S13 and S14 in Supplementary Materials).

**Figure 4:**
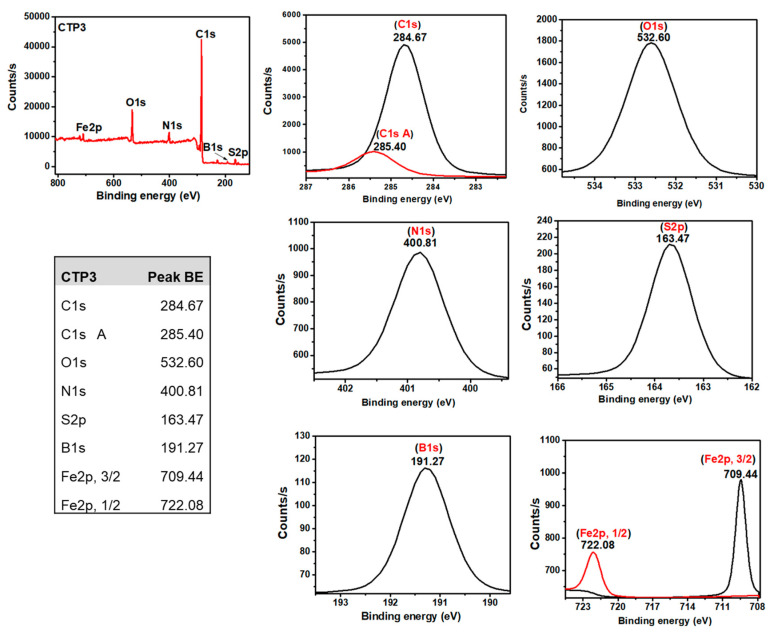
High-resolution XPS survey scan and spectra of C1s, O1s, N1s, S2p, B1s, and Fe2p of copolymer CTP3.

X-ray photoelectron spectroscopy (XPS) survey-scan spectra of CTP1-3 confirm the presence of all its constituting elements such as C1s, O1s, N1s, S2p, B1s, and Fe2p binding energies in the ranges ~284.6–285.4, 532.5–532.6, 400.7–400.8, 163.4–163.8, 191.1–191.2, and 709.3–722.2 eV, respectively (Figure 4, and Figures S18 and S19 in Supplementary Materials).

### 3.3. Synthesis of Poly(vinylene sulfone) Derivatives CTP4-6

**Scheme 3:**
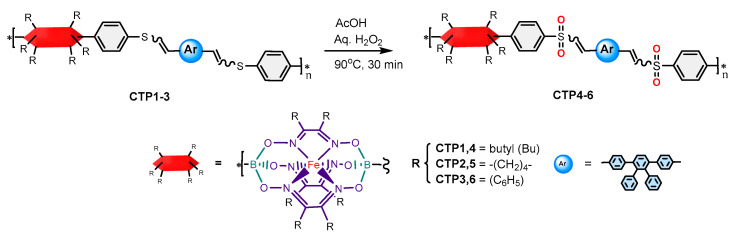
Synthesis of the poly(vinylene sulfone) copolymers CTP4-6.

Selective oxidation of poly(vinylene sulfide) copolymers CTP1-3 into their corresponding poly(vinylene sulfone) derivatives CTP4-6 was carried out using hydrogen peroxide in acetic acid (Scheme 3).

**Figure 5:**
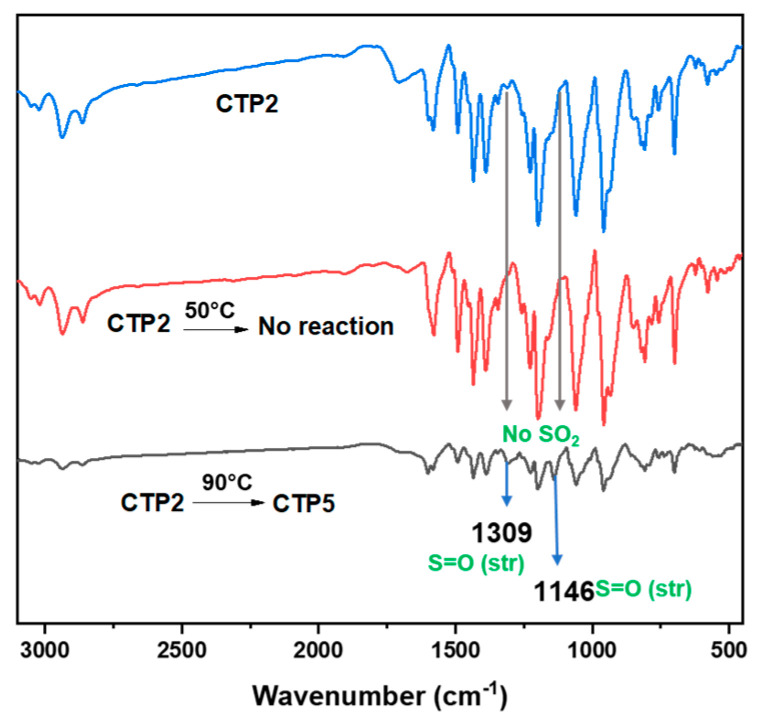
Comparative FTIR spectrum of CTP2 (**upper**), reaction of CTP2 at 50 °C (**middle**), and reaction of CTP2 at 90 °C (**lower**).

Nevertheless, control studies of the reaction temperature and time were carried out to optimize the selective oxidation conditions. Thus, a thorough analysis of the reaction mixture was monitored by FTIR, which is the best analytical technique to identify sulfone peaks. Surprisingly, only the starting materials were isolated when the reaction was carried out using the conventional conditions of 30 °C for 60 min and the same result was encountered when the temperature was raised to 50 °C (Figure 5 and Table 2 entry 1 and 2). On the other hand, decomposition products of copolymer CTP1 were detected upon increasing the reaction temperature of the medium to 100 °C (Table 2, entry 3). Interestingly, the desired target copolymer CTP4 was obtained when the temperature of the reaction medium was maintained at 90 °C for only 10 min of reaction time (Figure 5, and Table 2 entry 4). The poly(vinylene sulfone) copolymer derivatives CTP2-3 were oxidized into their respective poly(vinylene sulfone) moieties CTP5-6 at 90 °C but for a longer reaction time of 60 min (Figure 5 and Table 2 entry 5 and 6) and which could be possibly explained by the insolubility of synthons CTP2-3. The target copolymers CTP4-6 were isolated by simple filtration from the reaction medium in high yields (93–98%). FTIR absorption spectra of CTP4-6 depict the characteristic asymmetric and symmetric stretching vibrations peaks of sulfone (O=S=O) in the ranges ~1309–1316 cm^−1^ and ~1130–1146 cm^−1^, respectively (Figure 5, and Figures S15 and S17 in Supplementary Materials). It should be also noted that none of the poly(vinylene sulfone) derivatives CTP4-6 showed any FTIR absorption peak that can be attributed to the sulfoxide group, usually observed at ~1030 cm^−1^ [53].

**Figure 6:**
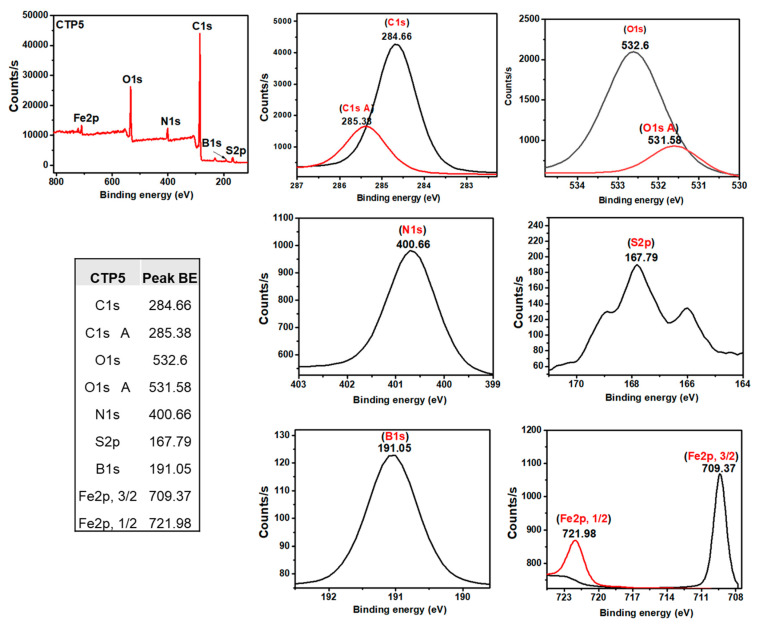
High-resolution XPS survey scan and spectra of C1s, O1s, N1s, S2p, B1s, and Fe2p of copolymer CTP5.

**Figure 7:**
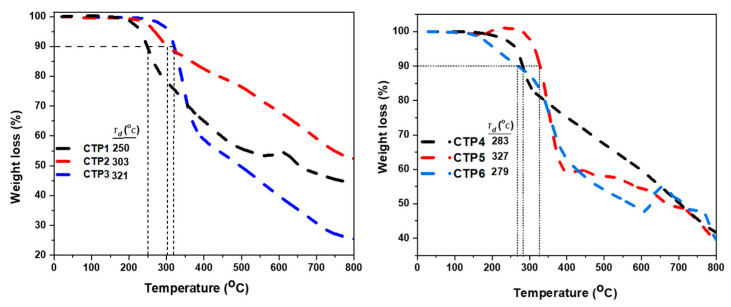
TGA thermograms of polymers CTP1-3 (**left**) and CTP4-6 (**right**); T_d_ represents the temperature of 10% weight loss.

The solubility of poly(vinylene sulfone) copolymer CTP4 permitted its structure elucidation by ^1^H- and ^13^C-NMR spectroscopy and its molar mass determination using GPC analysis (Figures S4, S8 and S9 in Supplementary Materials). Additionally, target copolymers CTP4-6 were all characterized by XPS and FTIR spectroscopy, which are the most suitable analytical techniques used to confirm the formation of sulfone groups. Additionally, the thermal properties of the poly(vinylene sulfone) copolymers were determined using TGA analysis (Figure 6 and Figure 7 and Figures S15–S17, S20 and S21 in Supplementary Materials).

XPS survey-scan spectra of CTP4-6 (Figure 6, and Figures S20 and S21 in Supplementary Materials) reveal the presence of all the constituting elements: namely, carbon, oxygen, nitrogen, sulfur, boron, and iron. Figure 6 portrays the XPS spectrum of CTP5 where the C1s peak is fitted into two main binding energy values at ~284.66 eV and 285.38 eV with the former assigned to the aromatic carbon groups (C=C) whereas the latter designated to the imine carbons (C=N). Similarly, the binding energy for oxygen exhibits two fingerprint peaks, one observed at ~531.58 eV, which corresponds to the sulfonic oxygen [56], and the second detected at 532.6 eV, which is assigned to the oxygen bonded to boron and nitrogen [40]. On the other hand, the N1s spectrum detected at 400.66 eV is correlated to the nitrogen (C–N). It is worthwhile to mention that the S2p binding energy in copolymer CTP2 detected at ~163.8 eV [57] was shifted to 167.79 eV [56], therefore, strongly confirming the conversion of the sulfide group in CTP2 into the sulfone in copolymer CTP5. B1s core-level spectrum was detected at 191.05 eV, which clearly divulges the presence of boron oxide (B–O). Figure 6 also reveals the XPS peak for Fe2p with binding energy values 709.37 eV and 721.98 eV, which are attributed to Fe(II)–N bonding order [58]. Similarly, target copolymers CTP4,6 portray conclusive XPS binding energy values that undoubtedly corroborate their formation (Figures S21 and S22 in Supplementary Materials).

Figure 7 illustrates the thermogravimetric analysis (TGA) of poly(vinylene sulfide) copolymers CTP1-3 depicting their 10% weight-loss temperatures in the range 250–321 °C, which corroborates the relatively high thermal stability of the target copolymers CTP1-3. Oxidation of the latter compounds into their respective poly(vinylene sulfone) moieties shows an increased thermal stability for CTP4,5 by ~30 °C, therefore, reaching 10% weight-loss temperatures of 283 and 327 °C, respectively, whereas the poly(vinylene sulfone)-bearing phenyl side groups CTP6 portrays a 10% weight-loss temperature of 279 °C, which is lower by 40 °C than its synthon CTP3 (Figure 7).

### 3.4. Gravimetric Iodine Capture Studies

**Figure 8:**
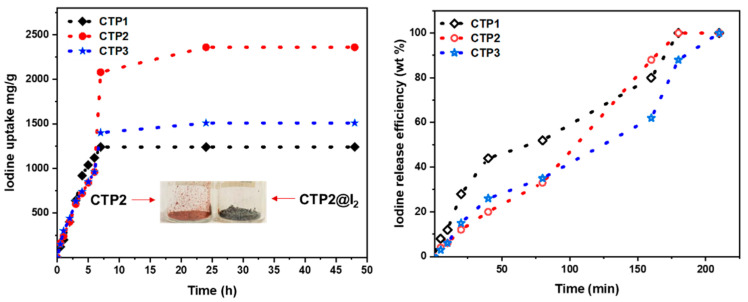
CTP1-3 gravimetric adsorption (**left**) at 80 °C and desorption (**right**) (heated at 120 °C in air) of iodine as a function of time. Inset: photographs showing the color change after iodine adsorption.

Copolymers CTP1-3 were tested as adsorbents of iodine vapor and their uptake capacity was evaluated using gravimetric analysis. A sample of a given copolymer and iodine flakes were placed in two connected closed pre-weighed vials and the container was degassed and maintained at 80 °C in an oven during the process. The mass of the copolymer was recorded at different time intervals until there was no further change (Figure 8). The results reveal that the iodine uptake gradually increased during the first ~24 h and reached a plateau thereafter. The target copolymers CTP1,3 revealed iodine uptake values 1240 and 1510 mg g^−1^, respectively, after 24 h of exposure to I_2_ vapors (Table 3, entry 1 and 3). Interestingly, ~960 mg g^−1^ of iodine vapor was adsorbed by CTP2 after seven hours, reaching a maximum iodine uptake of ~2360 mg g^−1^ after 48 h of exposure to gaseous I_2_ (Figure 8 and Table 3, entry 2). This result is considered promising when compared to some iodine adsorbents reported in the literature: namely, nitrogen-rich thorium–organic nanotube materials (955 mg g^−1^) [59], copper-loaded zeolites (450 mg g^−1^) [13], zeolitic imidazolate (ZIF-8) metalorganic frameworks MOFs (877 mg g^−1^) [60], porous carbon materials (955 mg g^−1^) [61], polyhedral silsesquioxane materials (363 mg g^−1^) [25], nitrogen-rich covalent organic framework (COFs, 980 mg g^−1^) [62], and copper- and zinc-based metal organic frameworks (Cu_2_O/Zn-MOF-NH_2_, 567 mg g^−1^) [63]. Although the iodine adsorption tests of poly(vinylene sulfone) copolymers CTP4-6 reveal relatively high uptakes reaching up to 1380 mg g^−1^, they suffer from needing a long exposure time to iodine vapors reaching up to 72 h, therefore displaying a lower performance when compared to CTP1-3 (Table 3 entry 5 and Figure S24 in Supplementary Materials). These findings corroborate the literature about the impeding effect that sulfones and sulfoxides generally have on iodine adsorption capacity [8].

**Figure 9:**
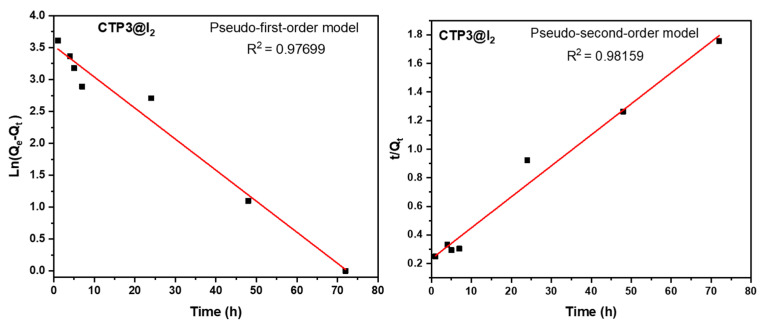
Kinetic modeling of iodine adsorption by CTP3 (gravimetric method) using pseudo-first-order kinetic model (**left**) and pseudo-second-order kinetic model (**right**).

The iodine adsorption kinetics of copolymers CTP1-3 were analyzed using pseudo-first-order and pseudo-second-order models (Figure 9, and Figures S22 and S23 in Supplementary Materials) [50]. The results show that the adsorption of CTP1 and CTP3 fit the pseudo-second-order kinetic model with linear correlation coefficient (R_2_) values of 0.997 and 0.981 (Figure 9, and Figure S22 in Supplementary Materials), respectively. On the other hand, CTP2 portrays a better correlation with the pseudo-first-order kinetic model with a linear correlation coefficient (R_2_) of 0.998 (Figure S23 in Supplementary Materials) [7,64].

**Figure 10:**
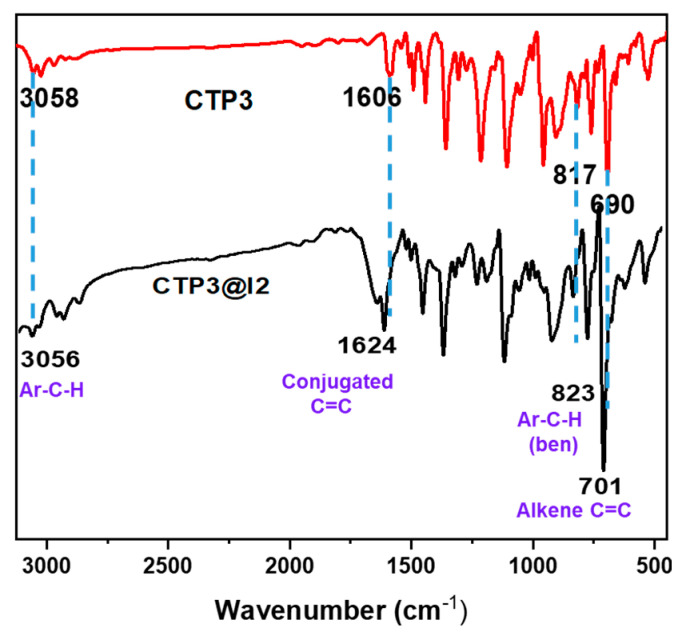
Comparative FTIR spectrum of CTP3 (**upper**) and iodine-loaded copolymers (CTP3@I_2_) (**lower**).

To better understand the mechanism of iodine capture by poly(vinylene sulfide) copolymers CTP1-3, a model sample of iodine-loaded copolymer (CTP3@I_2_) was investigated using FTIR spectroscopy. Figure 10 depicts the comparative FTIR spectra of copolymer CTP3 before and after iodine capture (CTP3@I_2_), clearly revealing the characteristic peaks shifts because of iodine absorption: mainly, the bands of aromatic C=C stretching, Ar-CH bending, and alkene C=C stretching vibrations. These conspicuous changes in the FTIR spectrum of CTP3@I_2_ strongly suggest the interaction between the abundant π-electrons of CTP3 and iodine species [29,50]. In addition, the presence of heteroatoms in the clathrochelate copolymer backbone, such as oxygen, nitrogen, and sulfur, can also plays a pivotal role in iodine capture by acting as binding sites for iodine molecules [65]. The minor change in the peaks also suggests weak interactions between the copolymer and iodine, thus suggesting a physisorption of the latter onto the surface of the copolymer.

**Figure 11:**
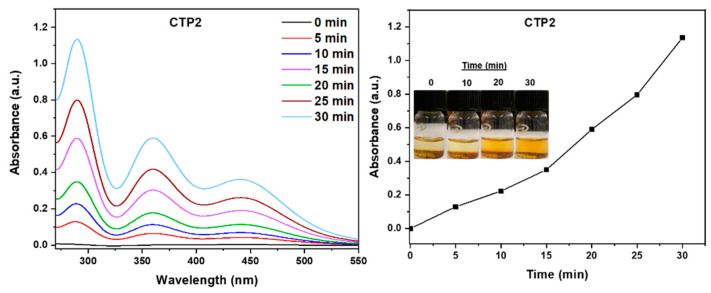
UV-Vis absorption spectra upon immersion of CTP2@I_2_ in ethanol. Inset: photos of the solutions showing the color change upon immersion in ethanol.

The complete desorption efficiency of the iodine-loaded copolymers (CTP1-3@I_2_) was recorded at different time intervals when adsorbed iodine by CTP1-3 was released by simple heating of the copolymers in air at 120 °C (Figure 8). Iodine desorption was further investigated by immersing an iodine-loaded copolymer sample CTP2@I_2_ in ethanol and recording the UV-Vis absorbance spectra at different time intervals (Figure 11). A noticeable increase in the intensity of the absorbance maxima that correspond to iodine was observed with time, which confirms the adsorbate release from CTP2 under ambient conditions. The amount of iodine released increased gradually and reached equilibrium after 30 min, and the color of the solution changed from colorless to yellow (Figure 11), which further confirms the iodine release in ethanol. These experimental observations strongly suggest that CTP2 can be employed as a sorbent material for efficient iodine vapor uptake.

**Figure 12:**
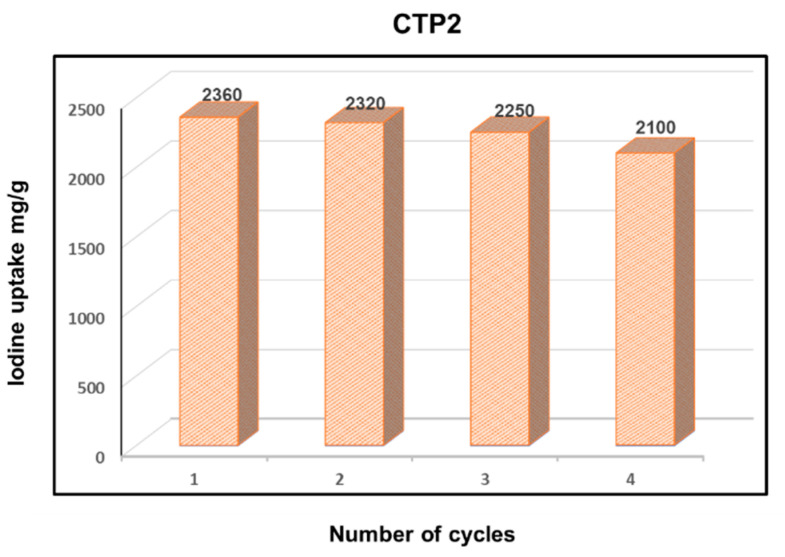
Graphical representation of iodine capture recyclability by CTP2.

The reusability of copolymers CTP1-3 was also investigated using the copolymer CTP2, which revealed the highest iodine uptake as a model adsorbent where a sample of CTP2 fully loaded with iodine vapors (CTP2@I_2_) was heated at 120 °C for 24 h, to ensure the complete release of the adsorbate from the copolymer backbone. The reactivated CTP2 was then exposed to iodine vapors and its uptake efficiency was recorded gravimetrically using the procedure described above. The regeneration test was repeated for four successive adsorption–desorption cycles, disclosing a minor change in the iodine adsorption efficiency for the regenerated copolymer CTP2 with a slight 11% decrease throughout the whole set of reusability experiments (Figure 12).

## 4. Conclusions

In summary, new metalorganic poly(vinylene sulfide) copolymers CTP1-3 bearing iron(II) clathrochelate unit and connected by tetraphenyl benzene vinylene sulfide groups were made in high yields via a simple one-pot reaction. Subsequently, the poly(vinylene-sulfide) copolymers CTP1-3 were selectively oxidized into their corresponding poly(vinylene-sulfone) copolymers CTP4-6. Iodine adsorption tests revealed uptake properties of CTP1-3 in the range 1240–2360 mg g^−1^. Kinetic studies of iodine adsorption by copolymers CTP1-3 reveal that the CTP1,3 fit the pseudo-secondary kinetic model whereas CTP2, which discloses the highest iodine uptake, is in good agreement with the pseudo-first-order kinetic model. In addition, reusability tests of CTP2 divulged promising results with virtually no change in its iodine adsorption efficacy even after four adsorption–desorption cycles. The versatile synthesis of the metalorganic copolymers CTP1-6 reported herein and their excellent iodine uptake properties clearly emphasize the potential use of iron(II) clathrochelate as a modular building block for designing novel materials for applications in environmental remediation, particularly the capture of radioactive elements.

## Figures and Tables

**Table 1:** Summary of optimized reaction conditions of copolymers CTP1-3.

Entry	Product	Time in Days	C_M_^a^ [M]	Yield (%)	Mn g mol^−1^	Mw g mol^−1^	Đ
1	CTP1	1	5.0	67	3672	10,115	2.8
2	CTP1	2	5.0	30	8827	40,396	4.6
3	CTP1	3	2.5	45	3524	10,026	2.8
4	CTP1	3	1.0	52	4989	9682	1.9
5	CTP1	5	2.5	91	5381	9554	1.8
6	CTP2	5	2.5	93	insoluble	-	-
7	CTP3	5	2.5	90	insoluble	-	-

^a^: molar concentration ×10^−2^ of FeCl_2._

**Table 2:** Summary of optimized reaction conditions of copolymers CTP4-6.

Entry	Copolymer	T (°C)	Time (min)	Oxidized Copolymer	Yield %
1	CTP1	30	60	No reaction	-
2	CTP1	50	60	No reaction	-
3	CTP1	100	60	Decomposed	-
4	CTP1	90	10	CTP4	93
5	CTP2	90	30	CTP5	98
6	CTP3	90	30	CTP6	97

**Table 3:** Summary of iodine adsorption by copolymers CTP1-6.

Entry	Copolymer	I_2_ Adsorption (mg g^−1^) after 24 h
1	CTP1	1240
2	CTP2	2360
3	CTP3	1510
4	CTP4	1140 ^a^
5	CTP5	1380 ^b^
6	CTP6	500

^a^: 48 h, ^b^: 72 h.

## Data Availability

The raw/processed data required to reproduce these findings can be shared upon demand.

## Supplementary Materials

The following supporting information can be downloaded at: https://www.mdpi.com/article/10.3390/polym14183727/s1, Figures S1–S4 (^1^H-NMR spectra of 3, TBM, & CTP1,4), Figures S5–S8 (^13^C-NMR spectra of 3, TBM, & CTP1,4), Figure S9 (GPC of CTP4), Figure S10–S11 (EI-HRMS spectra of 3, TBM), Figure S12 (comparative FTIR of 3 and TBM), Figure S13–S17 (FTIR spectra of CTP2-6), Figure S18–S21 (XPS spectra of CTP1-2,4 & CTP6), Figure S22–S23 (Kinetic modelling of iodine adsorption by CTP1 & CTP2), Figure S24 (gravimetric iodine adsorption and desorption of CTP5).
