# Supplementary material for: Synthesis of Iron(II) Clathrochelate-Based Poly(vinylene sulfide) with Tetraphenylbenzene Bridging Units and Their Selective Oxidation into Their Corresponding Poly(vinylene sulfone) Copolymers: Promising Materials for Iodine Capture
Source: Polymers (Basel). 2022 Sep 7;14(18):3727. doi: 10.3390/polym14183727 (PMC9504420; doi:10.3390/polym14183727)
Supplement: Supplementary file 1 [file polymers-14-03727-s001.zip › polymers-1870962-supplementary.pdf]

Supporting Information

Synthesis of Iron(II) Clathrochelate-Based Poly(vinylene sulfide) with Tetraphenylbenzene Bridging Units and their Selective Oxidation into their Corresponding Poly(vinylene sulfone) Copolymers: Promising Materials for Iodine Capture

Noorullah Baig[a,b], Suchetha Shetty[a,b], Sameh S. Habib[c], Ali A. Husain[c], Saleh Al-Mousawi[*c], Bassam Alameddine[*a,b]

[a] Department of Mathematics and Natural Sciences, Gulf University for Science and Technology, Kuwait.

[b] Functional Materials Group – CAMB, GUST, Kuwait.

[c] Department of Chemistry, University of Kuwait, Kuwait.

Tel: +965 2530 7476.

E-mail address: salehalmousawi@hotmail.com (S.A.-M.); alameddine.b@gust.edu.kw (B.A.)

Contents

Synthesis of **2 & 3**	(i), (ii)
^{1}H NMR spectrum of **3, TBM, & CTP1,4**	Figure S1-S4
^{13}C NMR spectrum of **3, TBM, & CTP1,4**	Figure S5-S8
GPC of **CTP4**	Figure S9
EI-HRMS of **3, TBM**	Figure S10-S11
Comparative FTIR of **3** and **TBM**	Figure S12
FTIR of **CTP2-6**	Figure S13-S17
XPS spectra of copolymer **CTP1-2,4 & CTP6**	Figure S18-S21
Kinetic modelling of iodine adsorption by **CTP1 & CTP2**	Figure S22-S23
Gravimetric iodine adsorption and desorption of **CTP5**	Figure S24

*†Corresponding author. *E-mail address*: salehalmousawi@hotmail.com (S.A.-M.); alameddine.b@gust.edu.kw (B.A.)

i). Synthesis of 2:

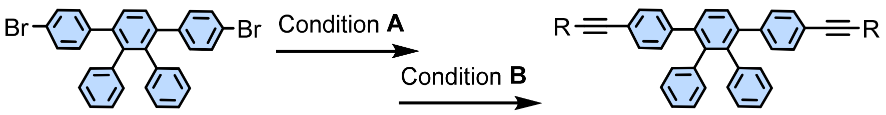

1 **2,3**

Condition **A:** $PdCl_2(PPh_3)_2$, TMS, CuI, TEA, Xylene, reflux

Condition **B:** THF, KOH, MeOH

2, R = $SiMe_3$

3, R = H

Scheme S1: Synthesis of **2&3**

3`,6`-bis(4-bromo)-1,1`:2`,1``-terphenylbenzene **1** (2.0 gm, 3.7 mmol), bis(triphenyl phosphine)palladium (II) dichloride $PdCl_2(PPh_3)_2$ (0.3 gm, 0.43mmol), copper (I) iodide CuI (0.14 gm, 0.74 mmol) and triphenyl phosphine PPh_3 (2.0 gm, 7.6 mmol) were dissolved in (3:1 volume ratio) triethylamine: xylene. Ethynyltrimethylsilane TMSA (6 gm, 61.2 mmol) was added, then kept for reflux 24 hours at 80-90°c with stirring which controlled by thin layer chromatography (TLC). The solvent was removed by evaporation under pressure. The product was purified by column chromatography (petroleum ether) to yield a yellowish precipitate; yield 75%; mp. 301-302 ºC; *Anal*. Calcd for $C_{40}H_{38}Si_2$: C, 83.57; H, 6.66. Found: C, 83.56; H, 6.64; EI-HRMS: *m*/*z* = 574.2506 (MH^+); $C_{40}H_{38}Si_2$ requires: *m*/*z* = 574.2507 (MH^+); ^1H - NMR (600 MHz, $CDCl_3$): δ= 0.249 (s,18H, CH_3), 6.78-6.80 (m, 4H, Ar-H), 6.94-6.95 (m, 6H, Ar-H), 7.06 (d,4H, *J*= 8.4 Hz, Ar-H), 7.27 (d, 4H, *J*= 8 Hz, Ar-H), 7.49 (s, 2H, Ar-H); 13C-NMR (150 MHz, $CDCl_3$): δ = 142.32, 140.64, 140.58, 139.62, 131.61, 131.46, 129.90, 129.46, 127.27, 126.03, 121.04, 105.31, 94.48, 0.15.

ii). Synthesis of 3:

3`,6`-bis(4-((trimethylsilane) ethynyl)-1,1`:2`,1``- terphenylbenzene **2** (1.6 gm, 2.8 mmol) was dissolved in THF (25 mL) and a solution of potassium hydroxide KOH (0.613 gm, 10.93 mmol) in methanol was added. The reaction was stirred at room temperature for 30 min which controlled by thin layer chromatography (TLC). The solvent was removed by evaporation under pressure. Desired product was isolated by column chromatography (petroleum ether) to yield a yellowish precipitate; yield 94.41%; mp. Up 400 ºC; *Anal*. Calcd for $C_{34}H_{22}$: C, 94.85; H, 5.15. Found: C, 94.87; H, 5.16; EI-HRMS: *m*/*z* = 430.1722 (MH^+); $C_{34}H_{22}$ requires: *m*/*z* = 430.1736 (MH^+); ^{1}H-NMR (600 MHz, DMSO-d_6): δ = 4.15 (s, 2H, ≡C−H), 6.82-6.83 (m, 4H, Ar-H), 6.95-6.96 (m, 6H, Ar-H), 7.10 (d, 4H, *J*= 8.4 Hz, Ar-H), 7.26 (d, 4H, *J*= 8 Hz, Ar-H), 7.50 (s, 2H, Ar-H)); ^{13}C-NMR (150 MHz, DMSO-d_6): δ = 141.89, 140.08, 139.87, 139.07, 131.00, 130.97, 129.75, 129.06, 126.97, 125.95, 119.67, 83.24, 81.01.

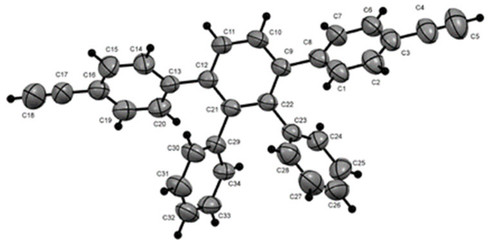

Single crystal structure of compound **3**

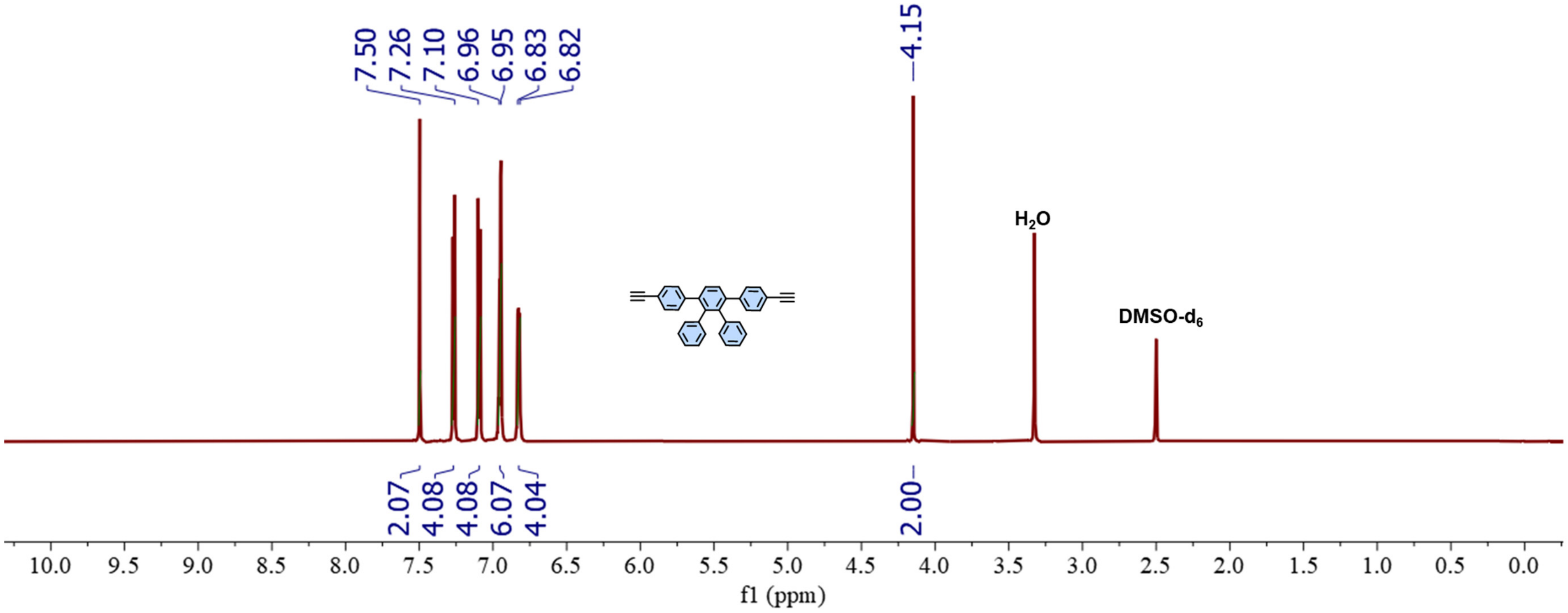

Figure S1: ^{1}H NMR spectrum of **3** (DMSO-d$_6$, 600 MHz)

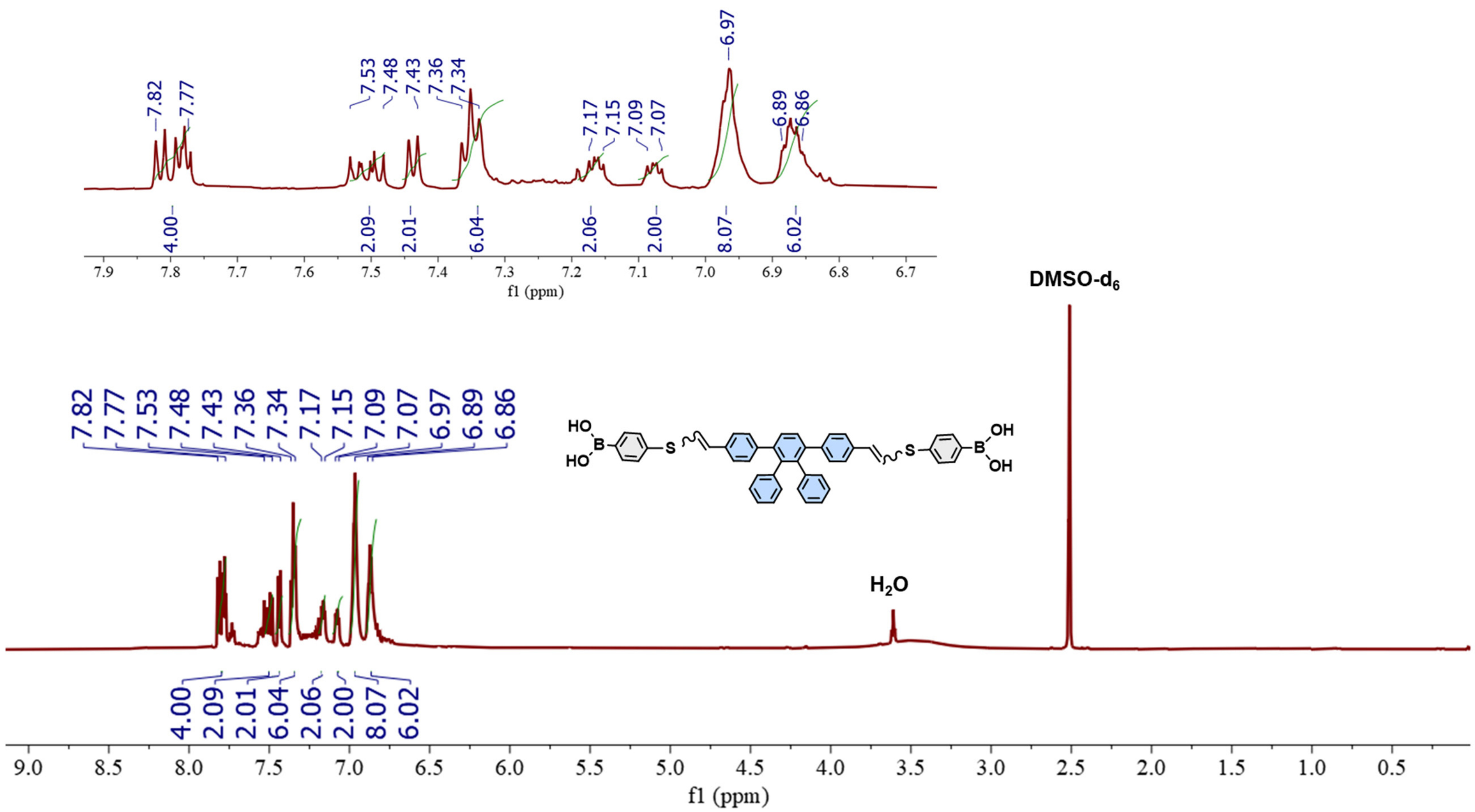

Figure S2: ^{1}H NMR spectrum of **TBM** (DMSO-d$_6$, 600 MHz)

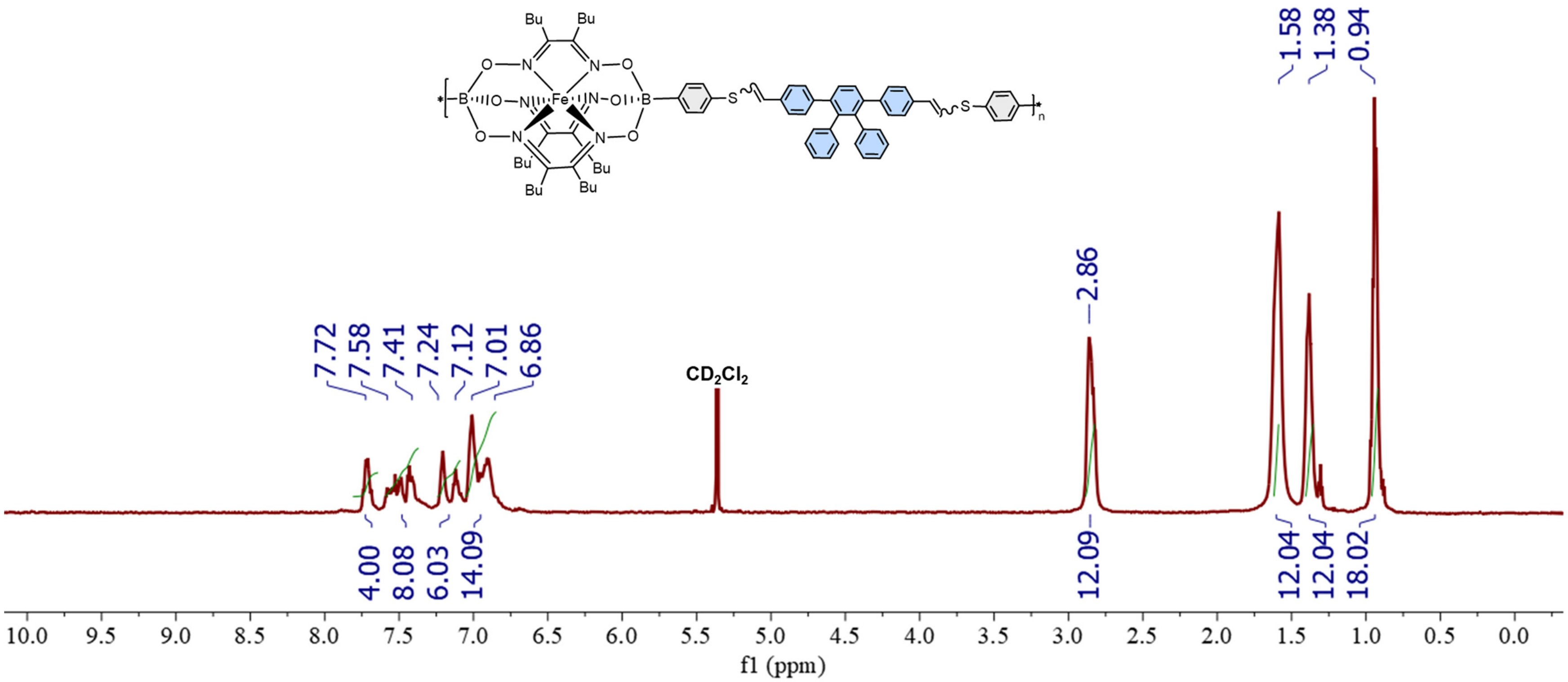

Figure S3: ^{1}H NMR spectrum of **CTP1** (CD_2Cl_2, 600 MHz)

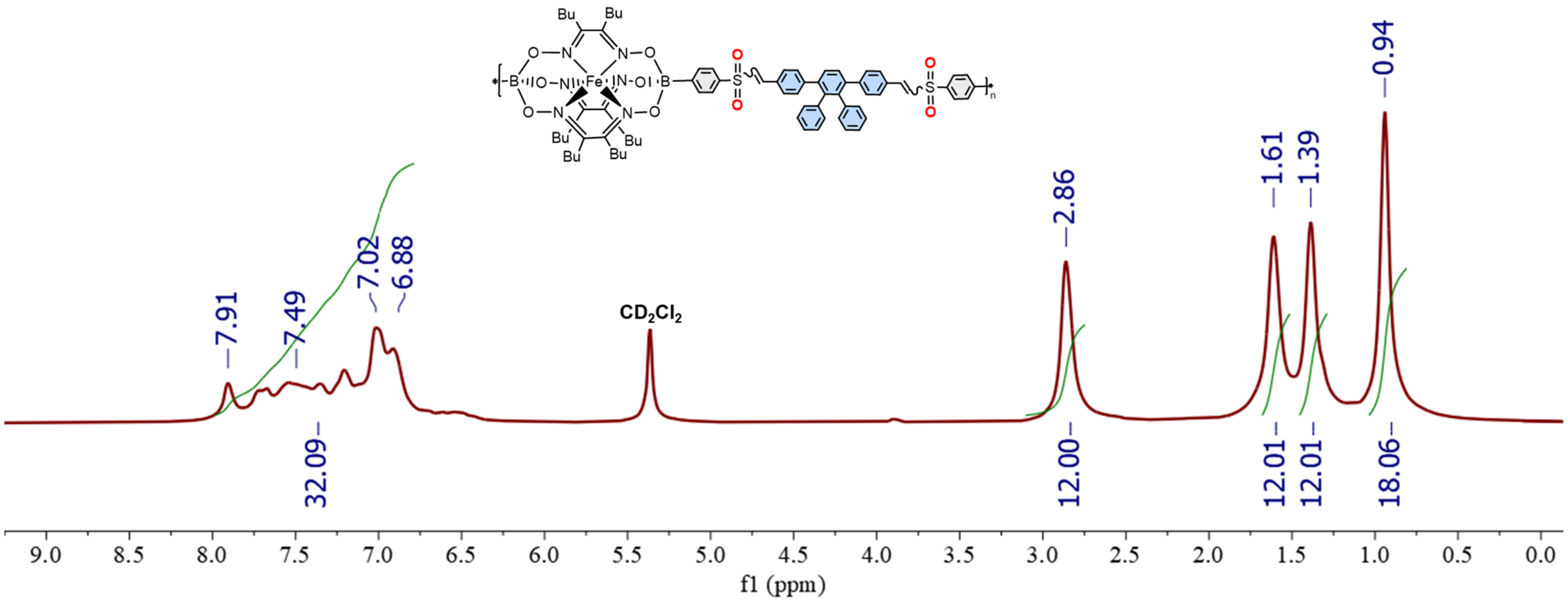

Figure S4: ^{1}H NMR spectrum of **CTP4** (CD_2Cl_2, 600 MHz)

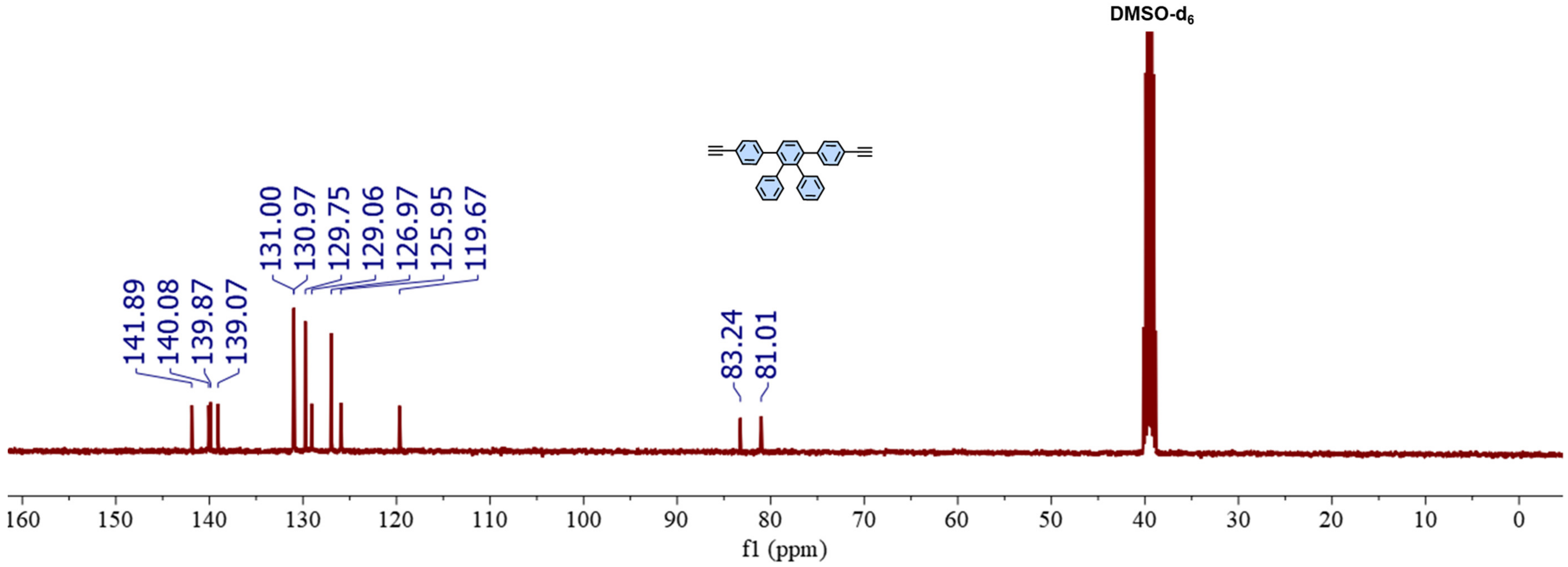

Figure S5: ${}^{13}C$ NMR spectrum of **3** (DMSO-d_6, 150 MHz)

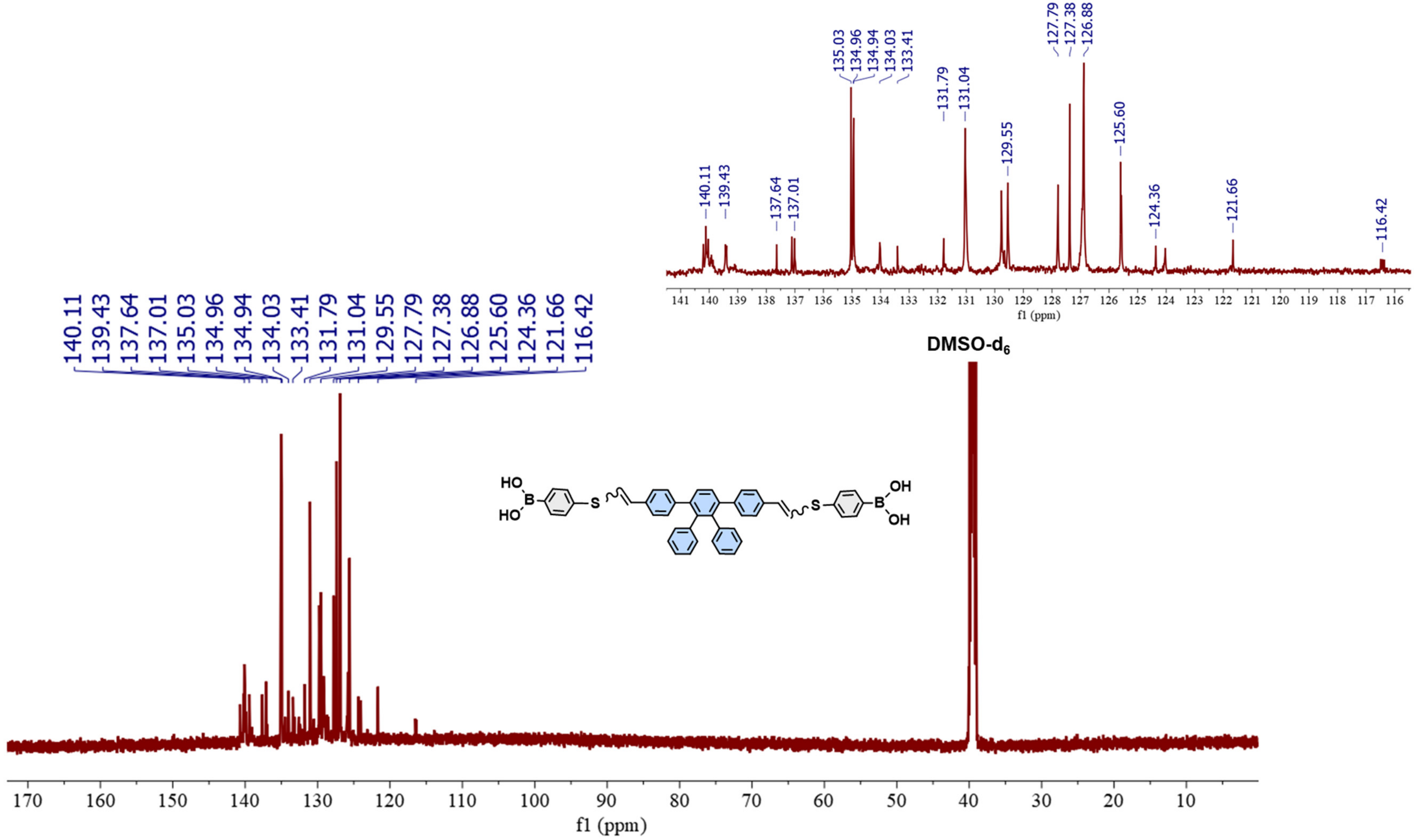

Figure S6: ^{13}C NMR spectrum of **TBM** (DMSO-d_6, 150 MHz)

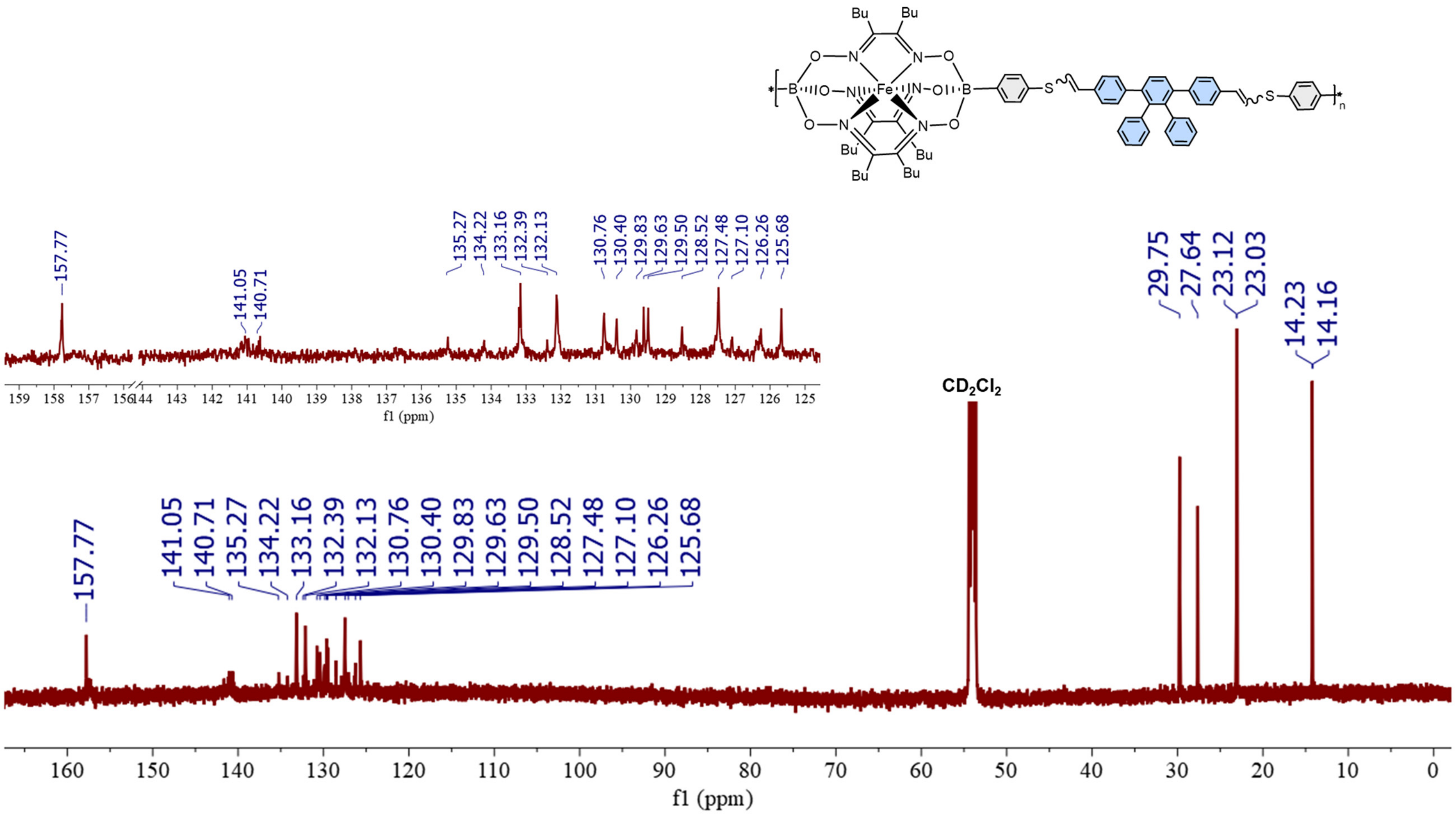

Figure S7: ^{13}C NMR spectrum of **CTP1** (CD_2Cl_2, 150 MHz)

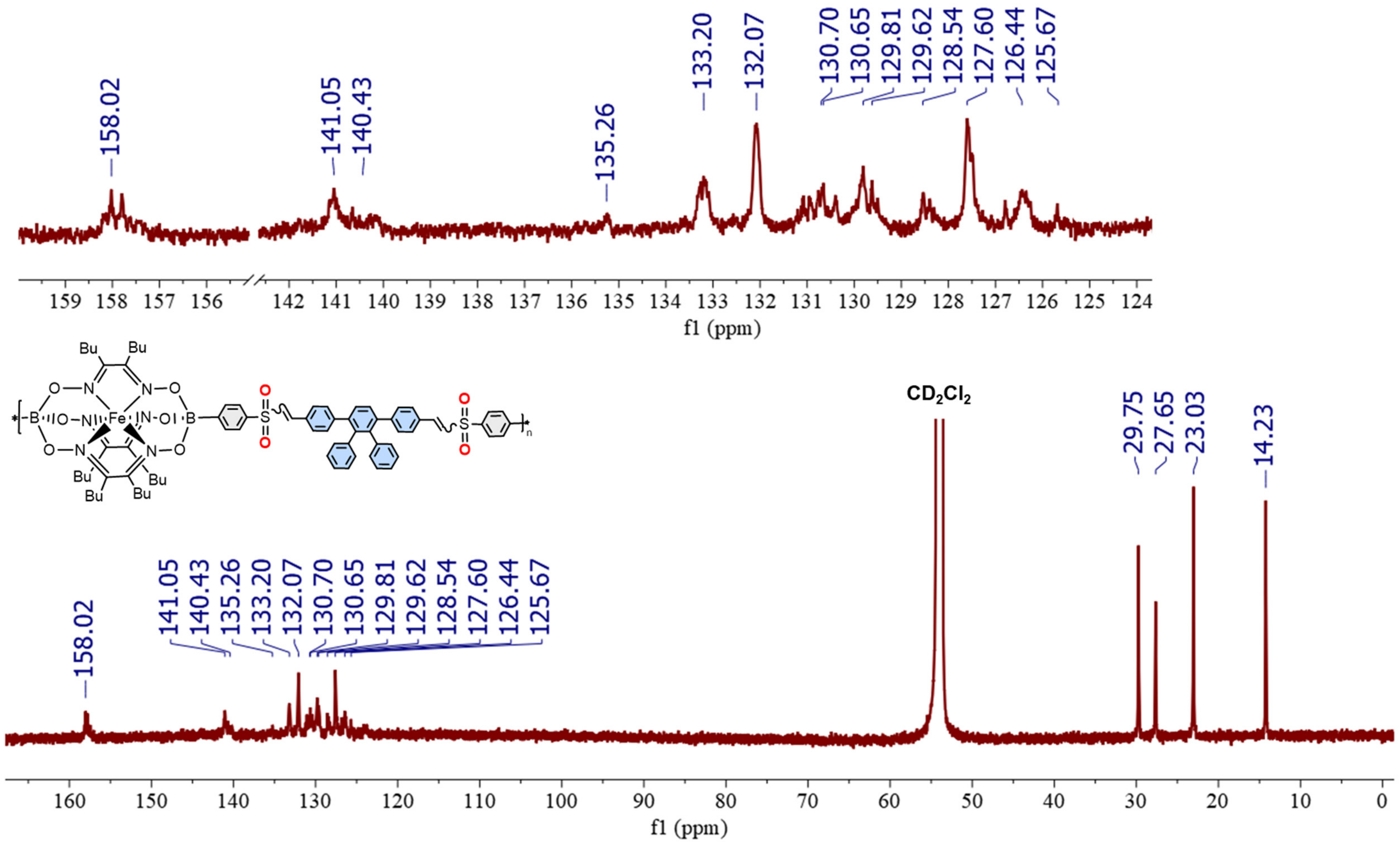

Figure S8: ^{13}C NMR spectrum of **CTP4** (CD_2Cl_2, 150 MHz)

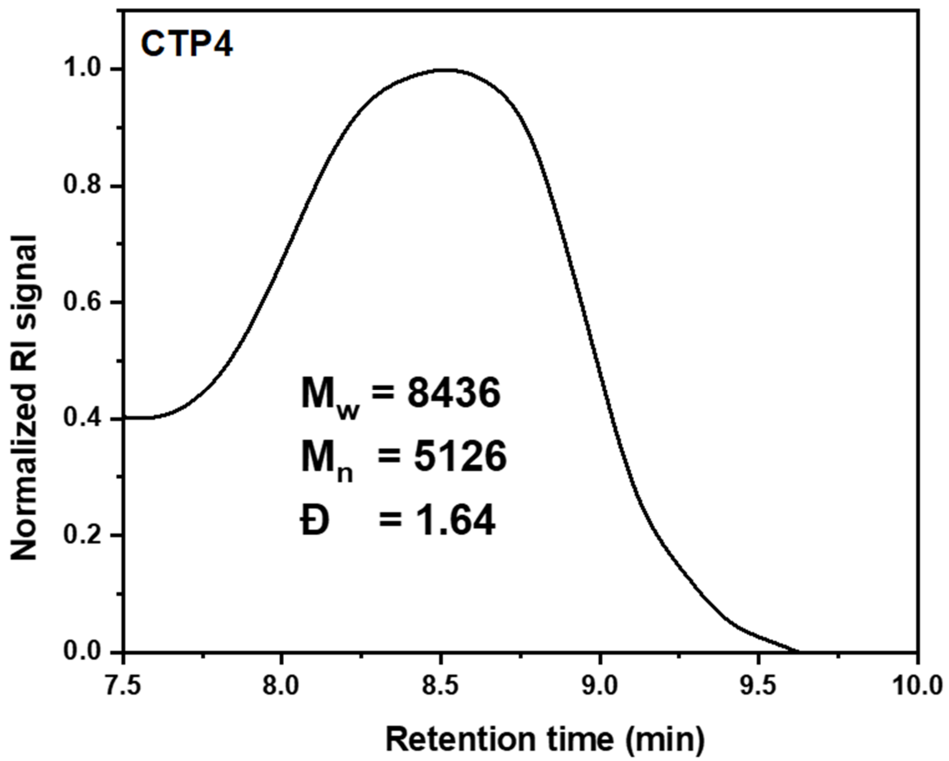

Figure S9: GPC of **CTP4**

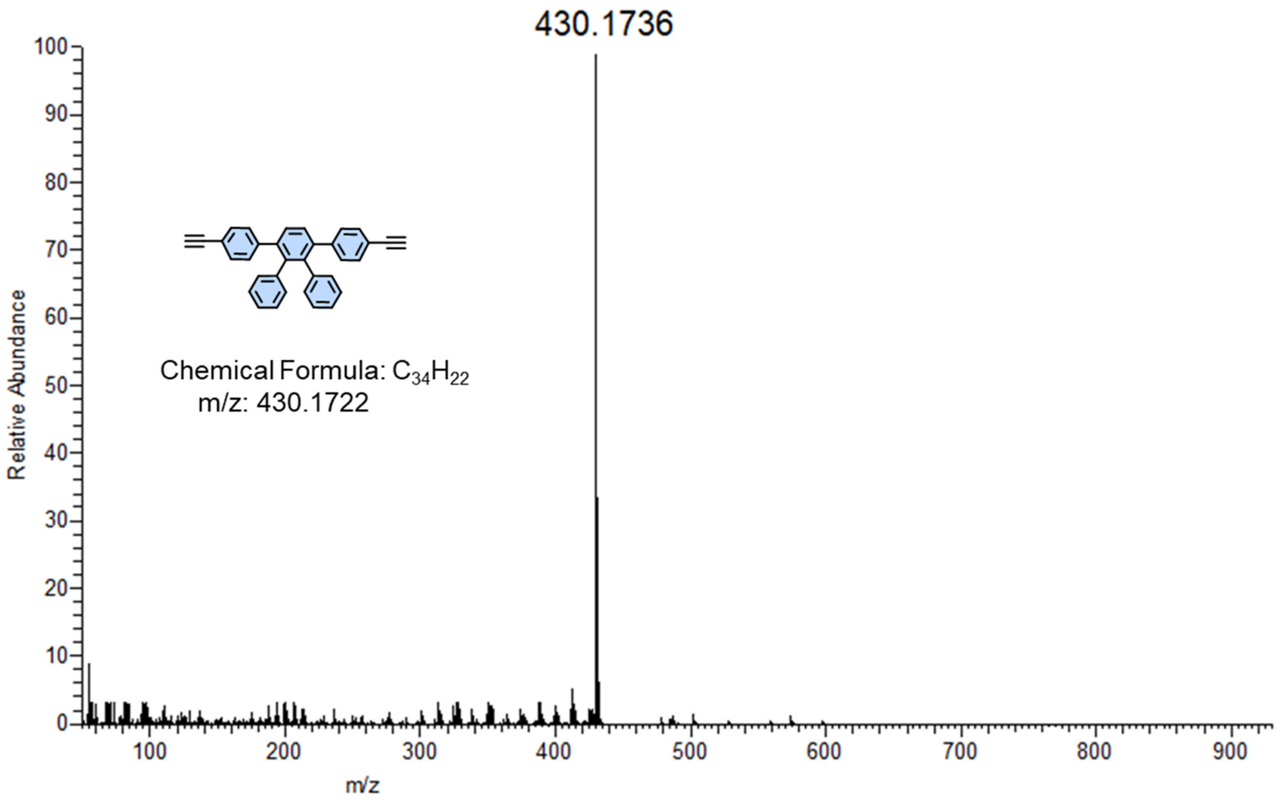

Figure S10: EI-HRMS of **3**

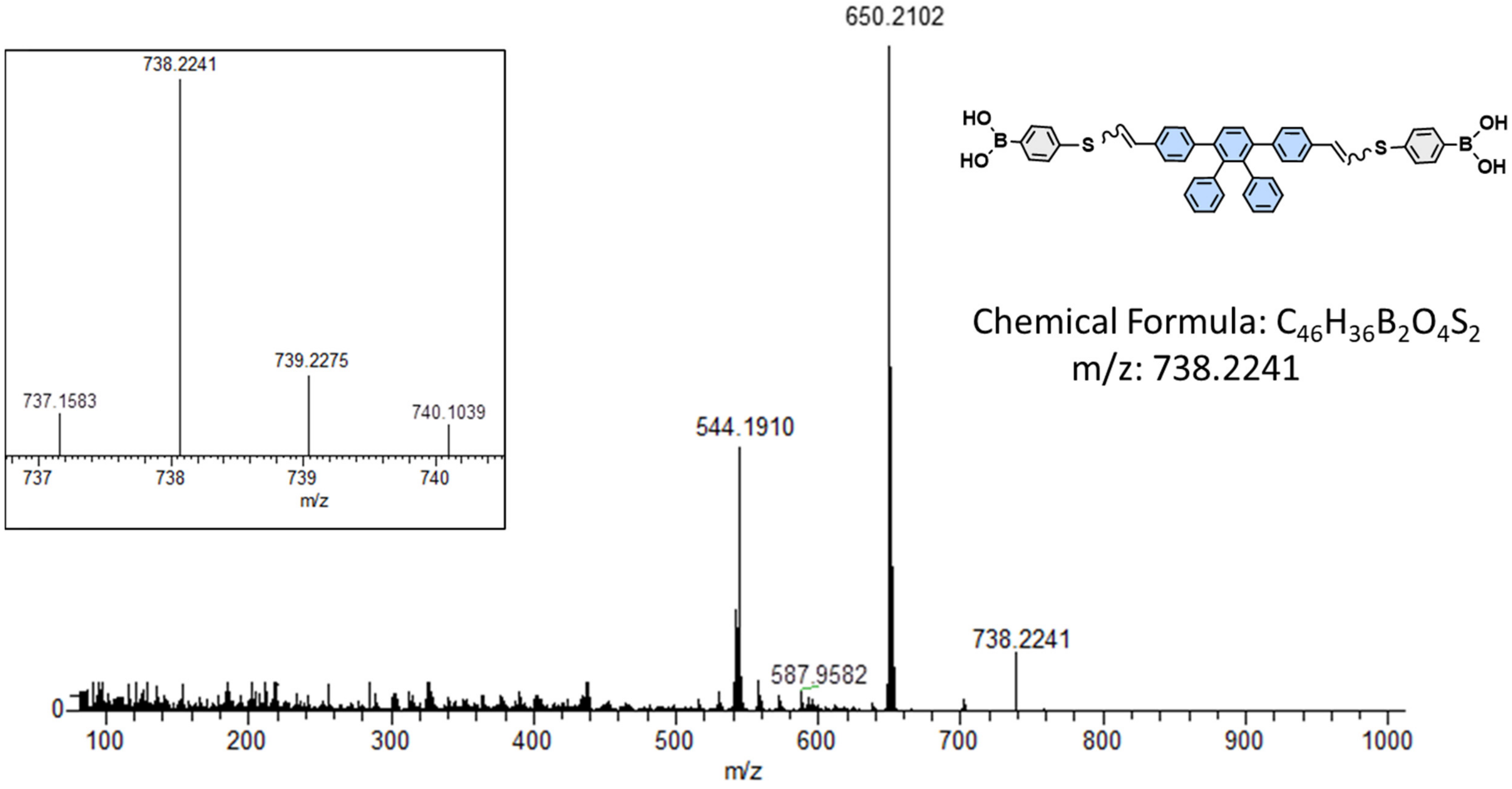

Figure S11: EI-HRMS of **TBM** (inset; isotopic distribution at m/z)

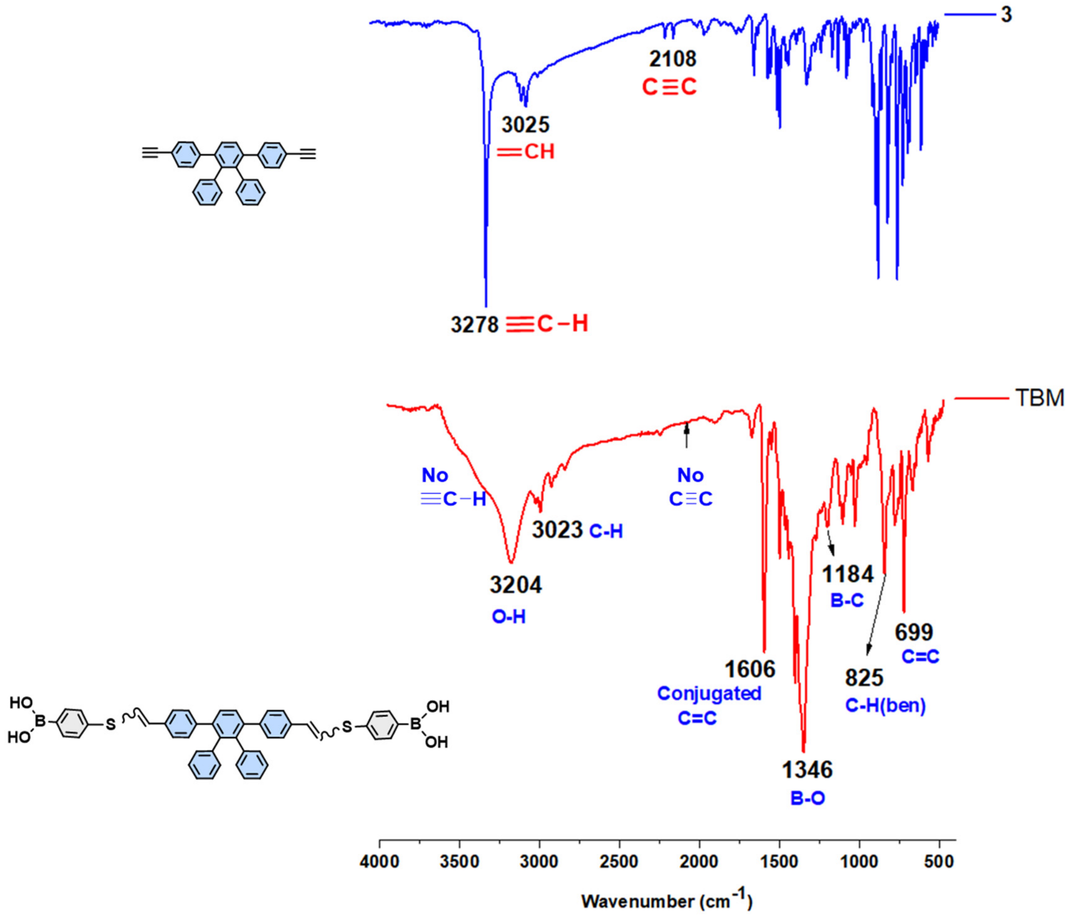

Figure S12: Comparative FT-IR spectrum of **3** (up) and **TBM** (down)

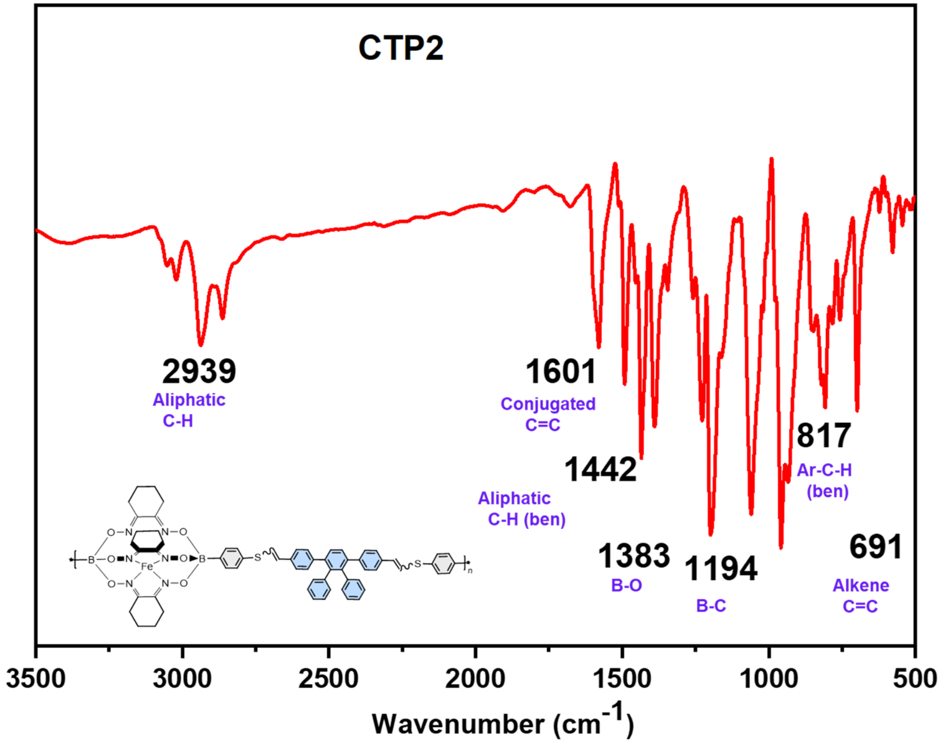

Figure S13: FT-IR spectrum of **CTP2**

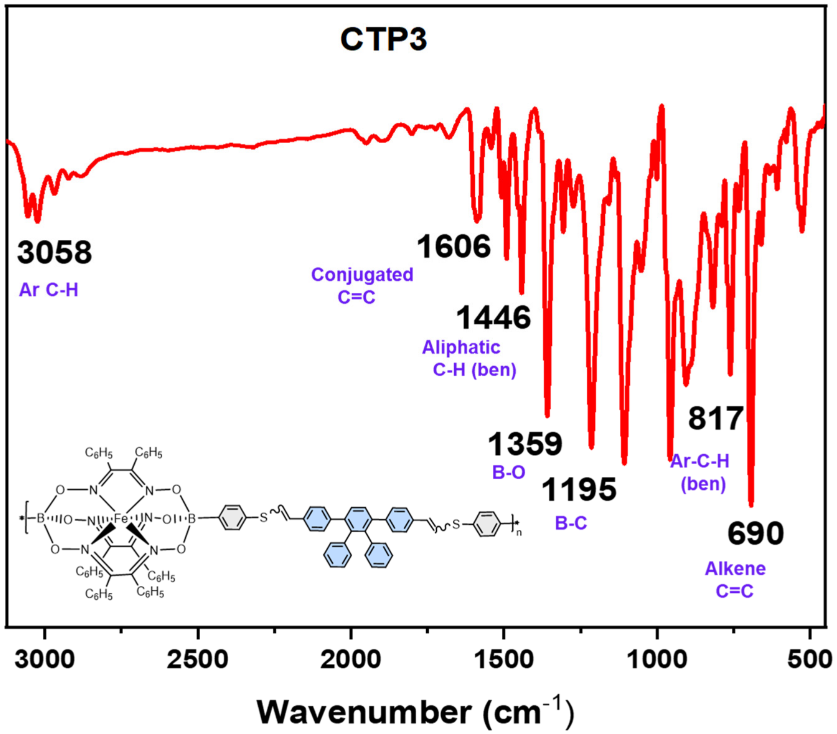

Figure S14: FT-IR spectrum of **CTP3**

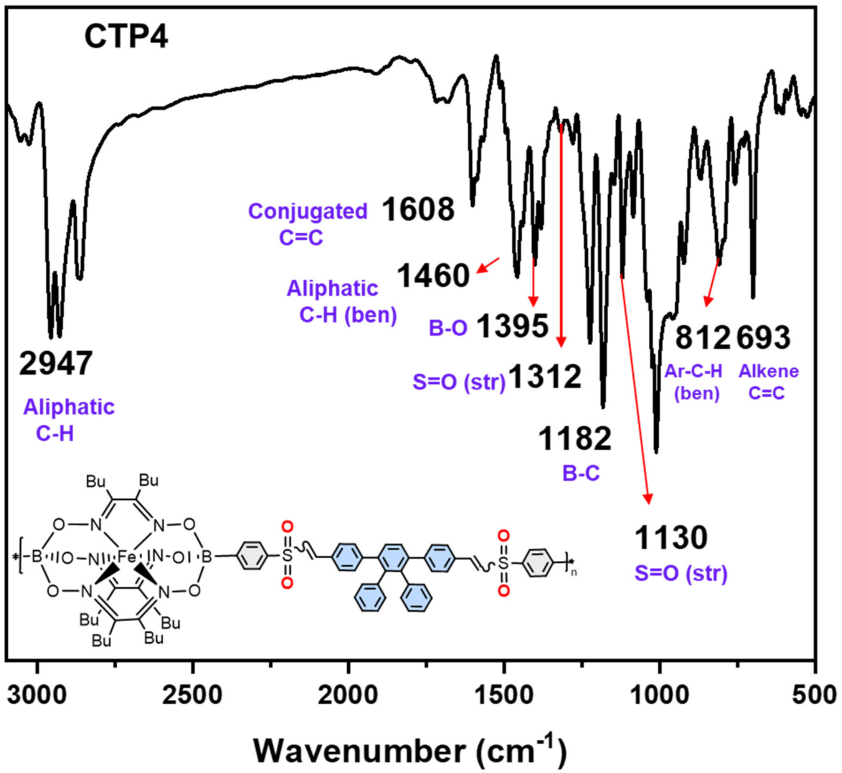

Figure S15: FT-IR spectrum of **CTP4**

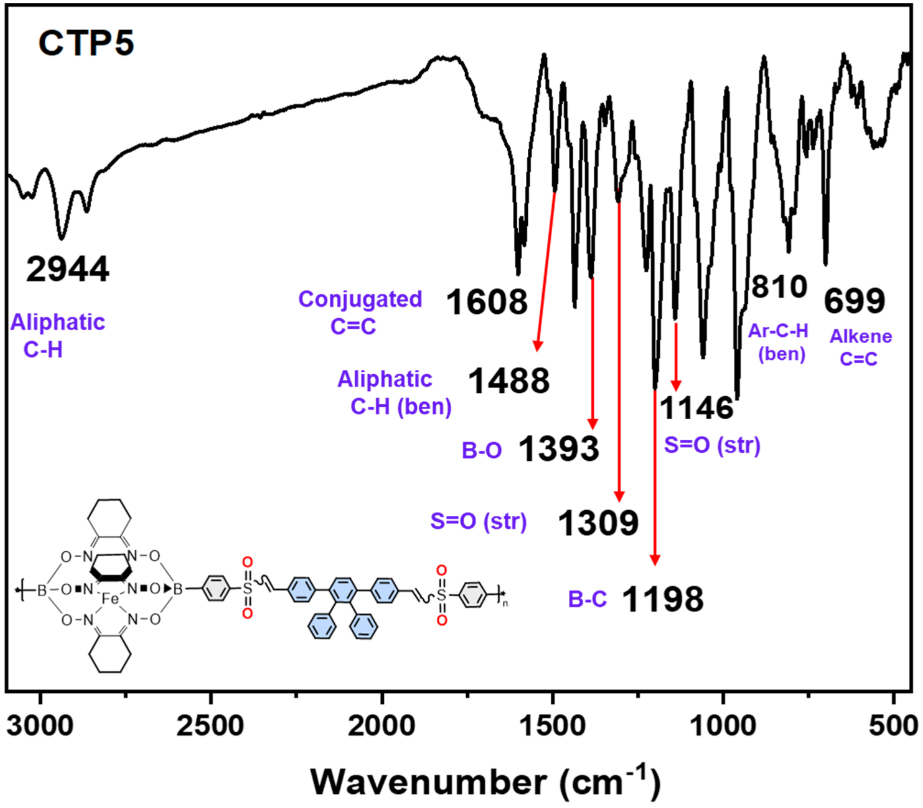

Figure S16: FT-IR spectrum of **CTP5**

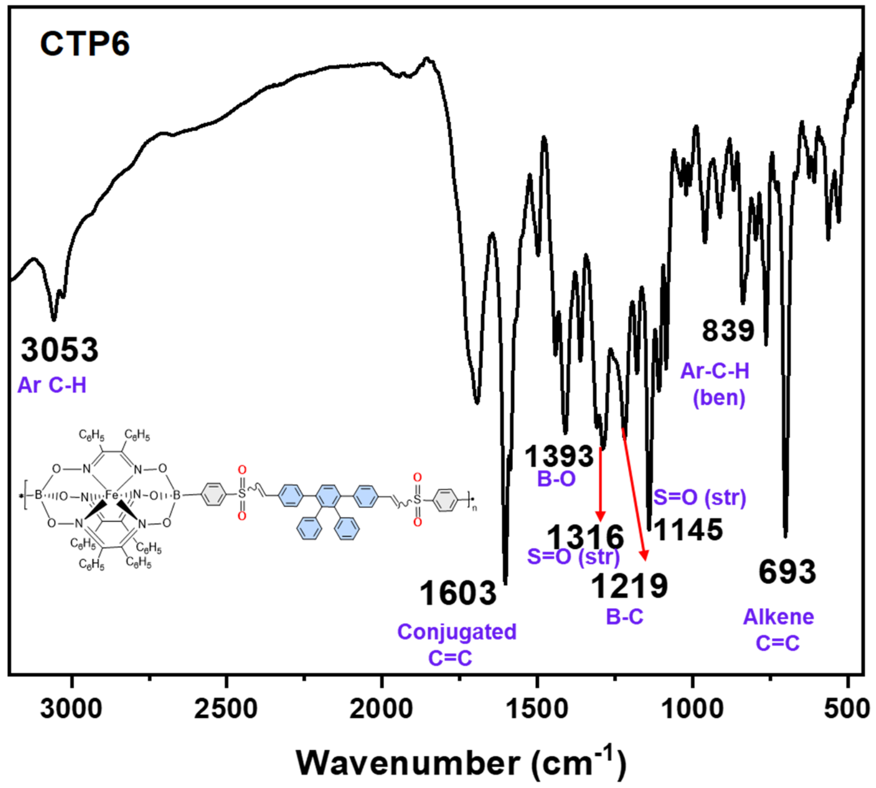

Figure S17: FT-IR spectrum of **CTP6**

CTP1	Peak BE
C1s	284.65
C1s A	285.31
O1s	532.5
N1s	400.75
S2p	163.63
B1s	191.12
Fe2p, 3/2	709.34
Fe2p, 1/2	721.9

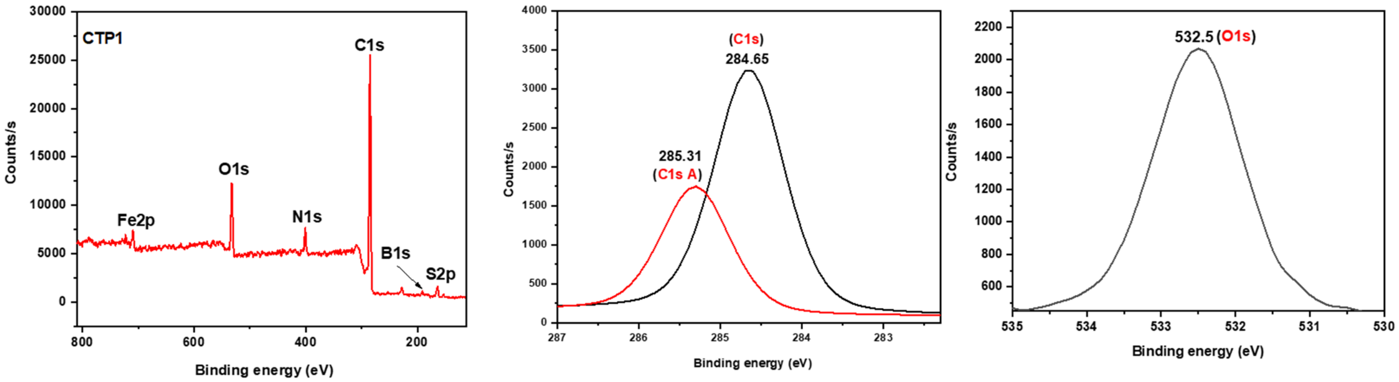

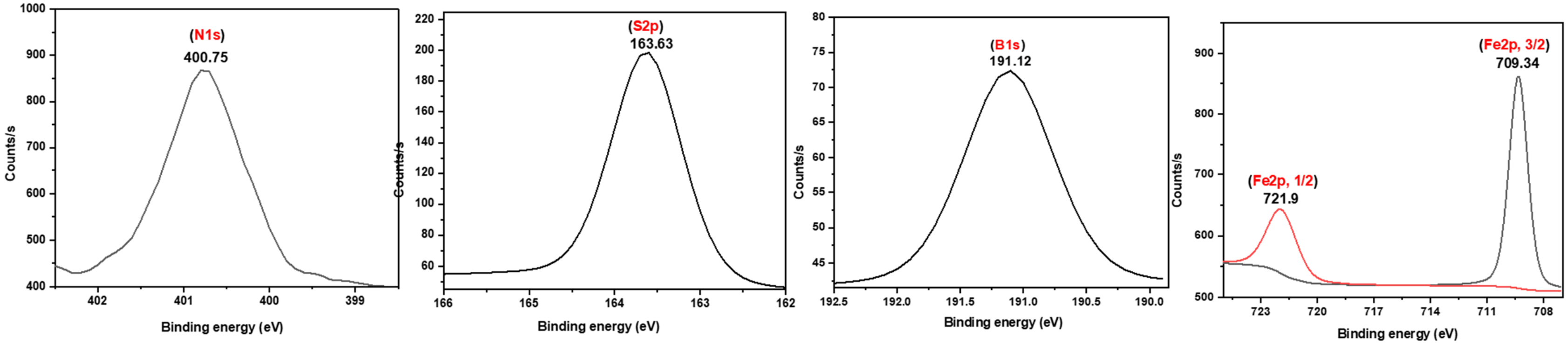

Figure S18: High-resolution XPS survey scan and spectra of C1s, O1s, N1s, S2p, B1s and Fe2p of copolymer **CTP1**

CTP2	Peak BE
C1s	284.65
C1s A	285.44
O1s	532.64
N1s	400.76
S2p	163.80
B1s	191.23
Fe2p, 3/2	709.48
Fe2p, 1/2	722.20

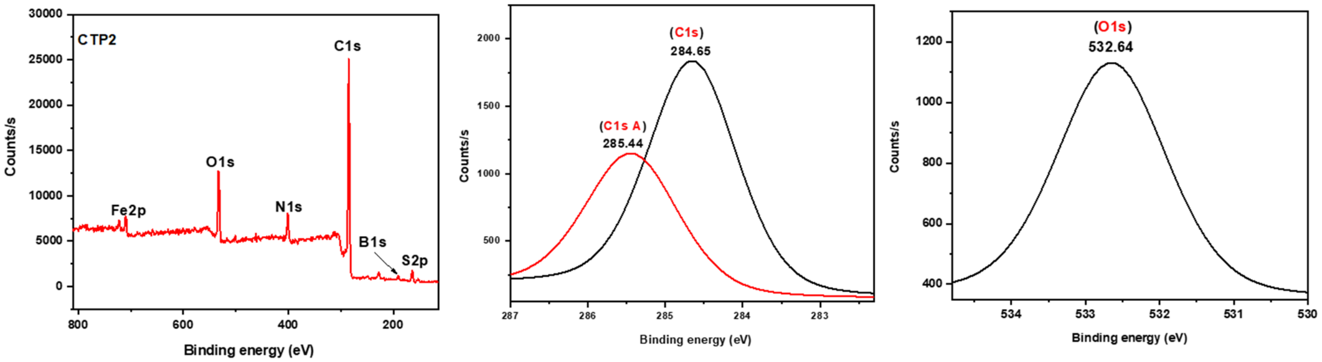

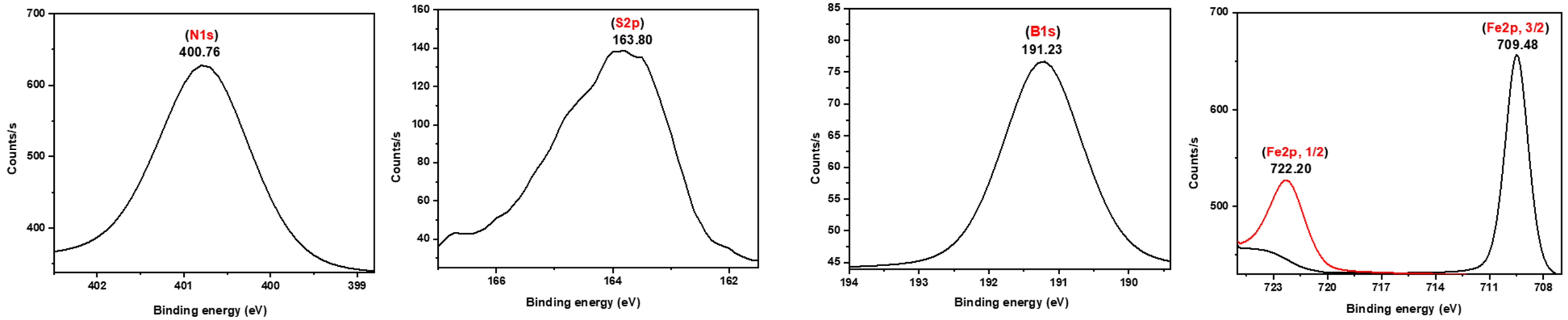

Figure S19: High-resolution XPS survey scan and spectra of C1s, O1s, N1s, S2p, B1s and Fe2p of copolymer **CTP2**

CTP4	Peak BE
C1s	284.66
C1s A	285.38
O1s	532.6
O1s A	531.58
N1s	400.66
S2p	167.79
B1s	191.05
Fe2p, 3/2	709.37
Fe2p, 1/2	721.98

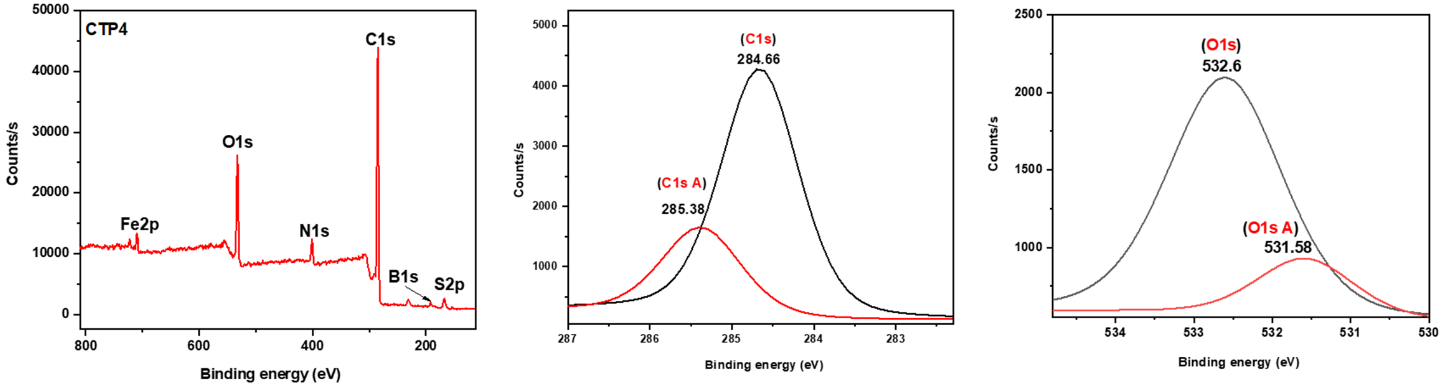

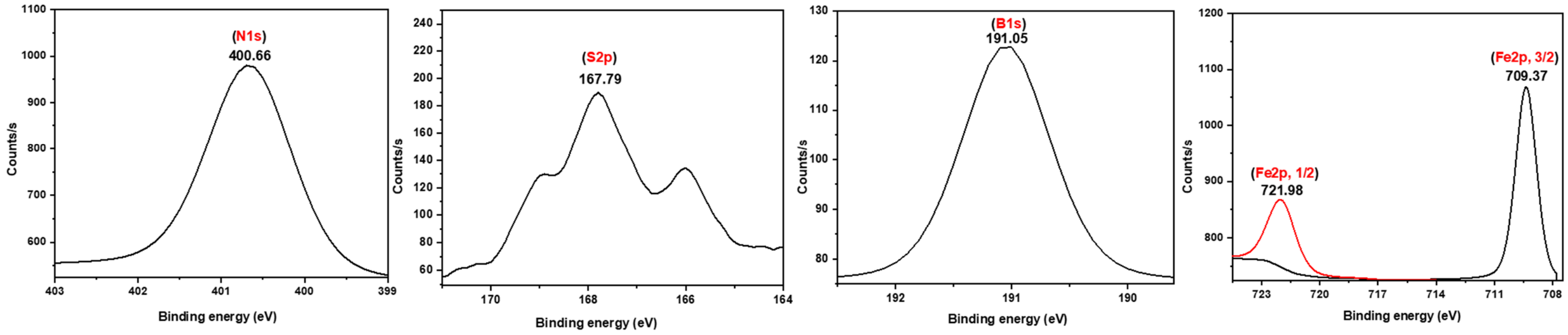

Figure S20: High-resolution XPS survey scan and spectra of C1s, O1s, N1s, S2p, B1s and Fe2p of copolymer **CTP4**

CTP6	Peak BE
C1s	284.69
C1s A	285.42
O1s	532.34
O1s A	533.42
N1s	400.94
S2p	168.19
B1s	191.43
Fe2p, 3/2	709.67
Fe2p, 1/2	722.26

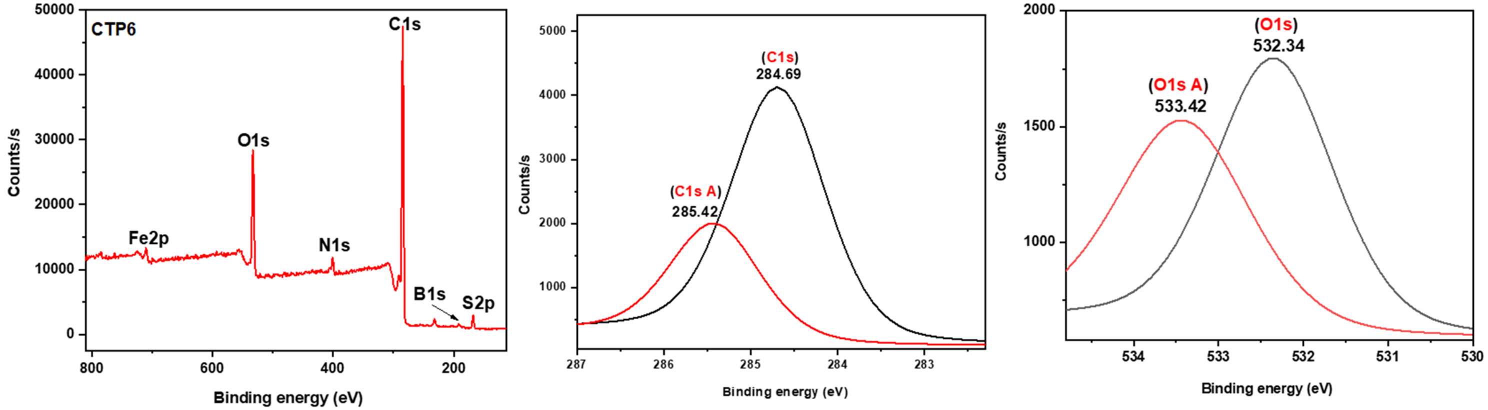

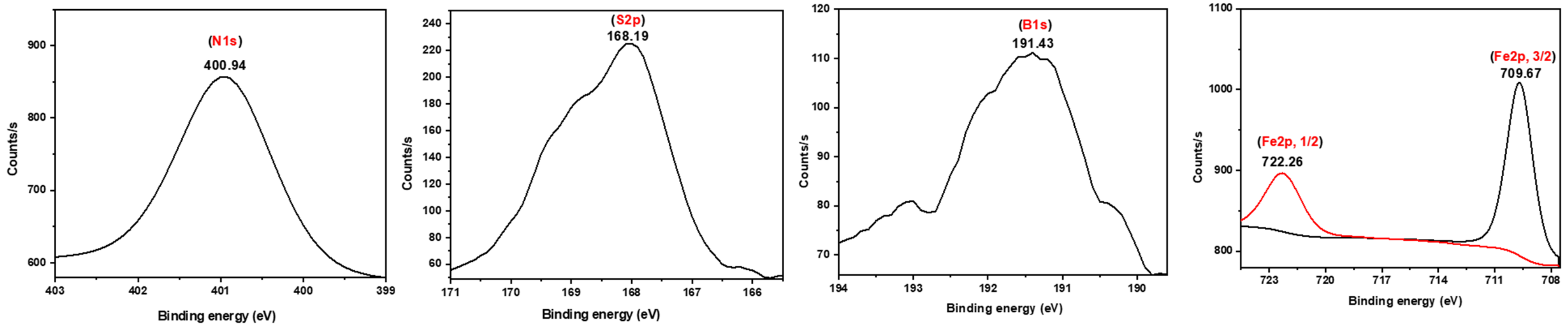

Figure S21: High-resolution XPS survey scan and spectra of C1s, O1s, N1s, S2p, B1s and Fe2p of copolymer **CTP6**

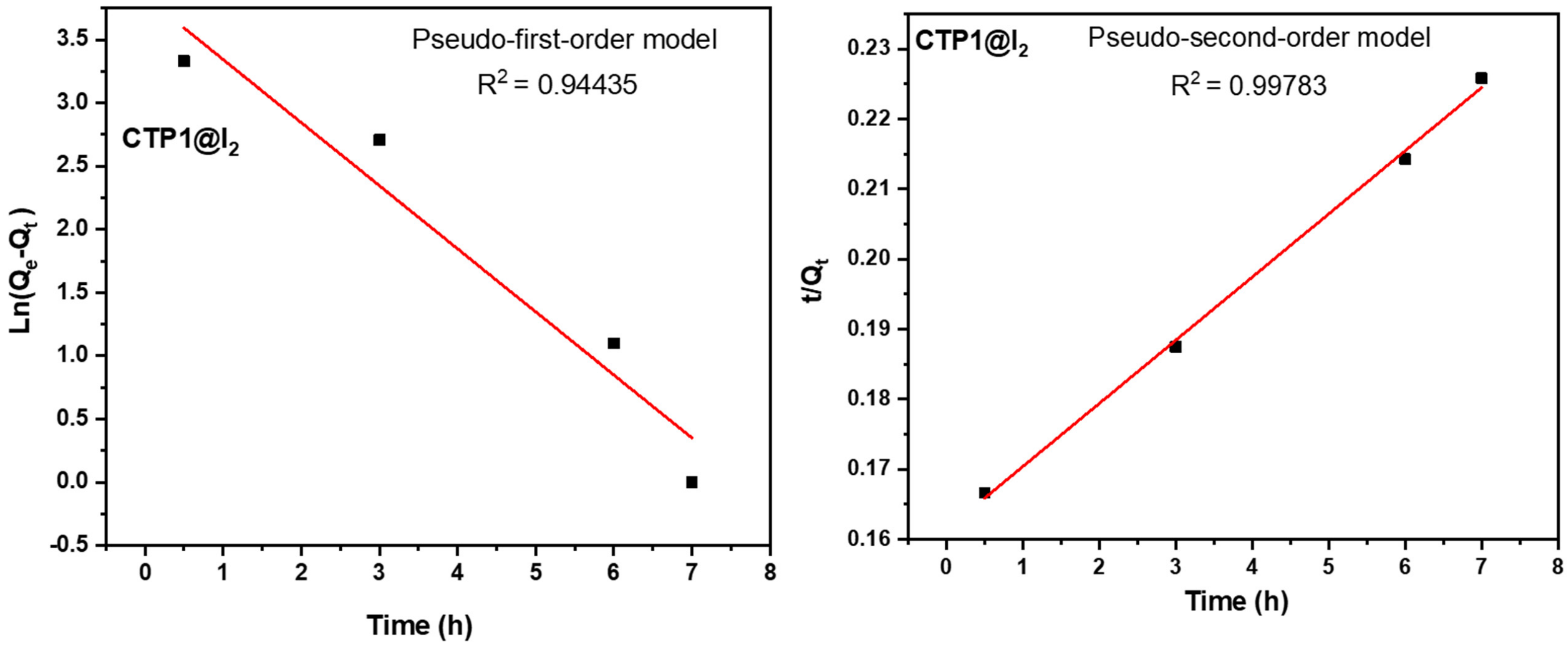

Figure S22: Kinetic modelling of iodine adsorption by **CTP1** (Gravimetric method) using pseudo-first-order kinetic model (left) and pseudo-second-order kinetic model (right)

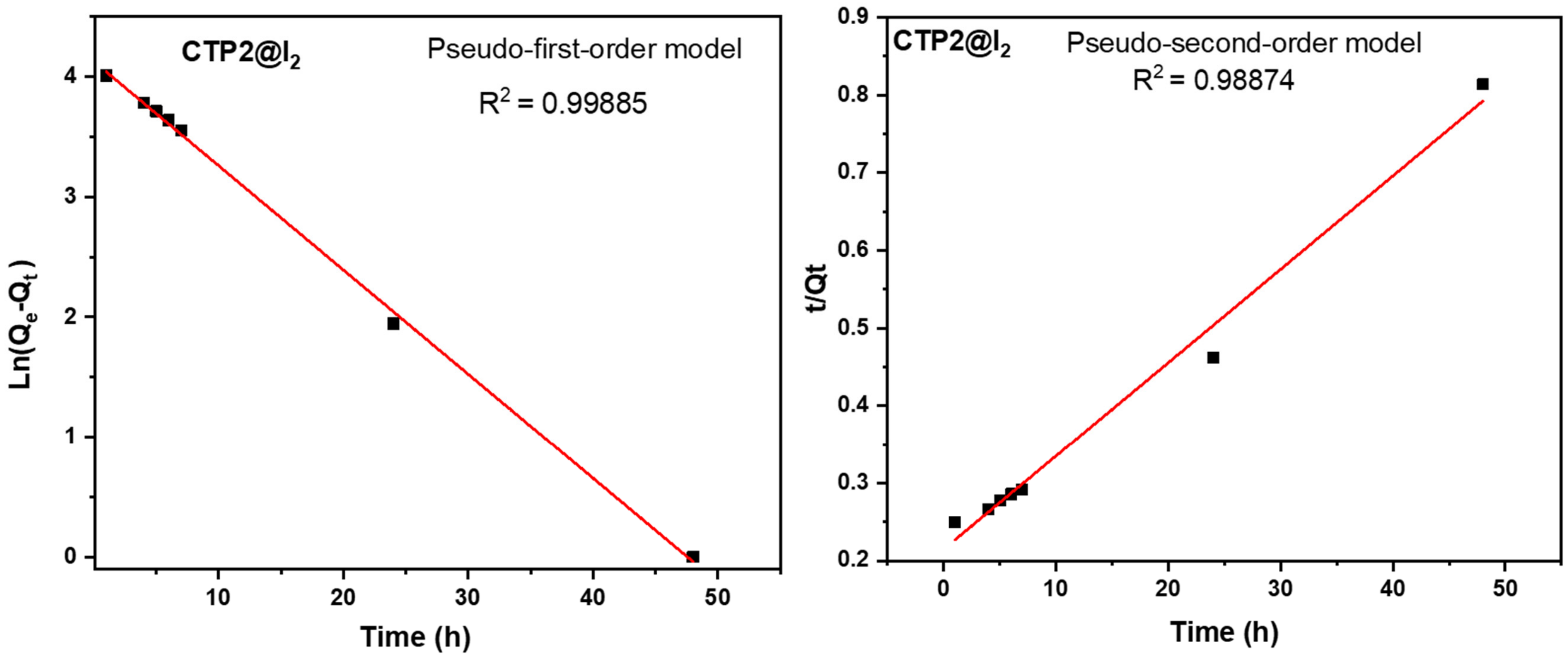

Figure S23: Kinetic modelling of iodine adsorption by **CTP2** (Gravimetric method) using pseudo-first-order kinetic model (left) and pseudo-second-order kinetic model (right)

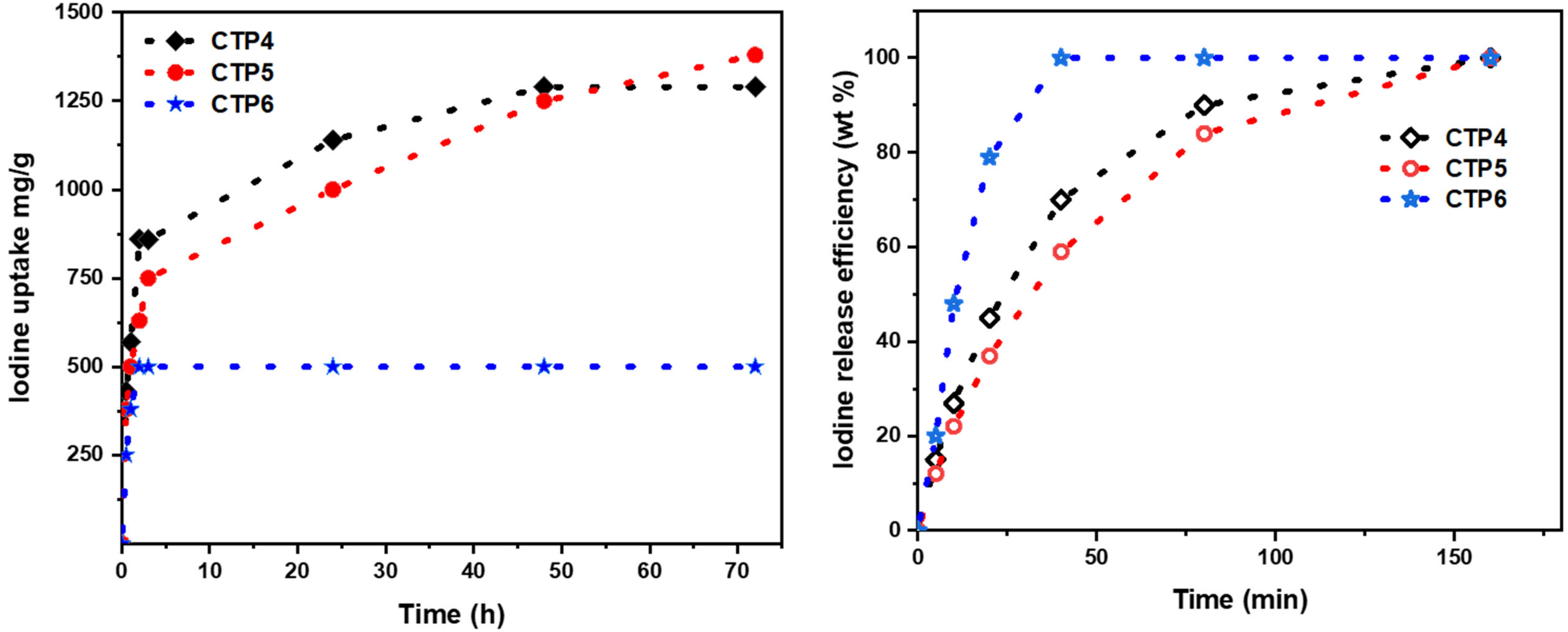

Figure S24: Gravimetric adsorption at 80°C (left) and desorption at 120°C (right) of iodine vapor
